# Integration of beef cattle international pedigree and genomic estimated breeding values into national evaluations, with an application to the Italian Limousin population

**DOI:** 10.1186/s12711-023-00813-2

**Published:** 2023-06-12

**Authors:** Renzo Bonifazi, Mario P. L. Calus, Jan ten Napel, Roel F. Veerkamp, Stefano Biffani, Martino Cassandro, Simone Savoia, Jérémie Vandenplas

**Affiliations:** 1 Animal Breeding and Genomics, Wageningen University & Research, P.O. Box 338, 6700 AH Wageningen, The Netherlands; 2 Istituto Di Biologia E Biotecnologia Agraria, Consiglio Nazionale Delle Ricerche, Via Edoardo Bassini 15, 20133 Milano, Italy; 3 Department of Agronomy, Food, Natural Resources, Animals and Environment (DAFNAE), University of Padova, Viale dell’Università 16, 35020 Legnaro, PD Italy; 4 National Federation of National Breeders Associations (FedANA), XXIV Maggio 43, 00187 Roma, Italy; 5 Interbull Centre, Department of Animal Breeding and Genetics, SLU-Box 7023, 75007 Uppsala, Sweden

## Abstract

**Background:**

International evaluations combine data from different countries allowing breeders to have access to larger panels of elite bulls and to increase the accuracy of estimated breeding values (EBV). However, international and national evaluations can use different sources of information to compute EBV (EBV_INT_ and EBV_NAT_, respectively), leading to differences between them. Choosing one of these EBV results in losing the information that is contained only in the discarded EBV. Our objectives were to define and validate a procedure to integrate publishable sires’ EBV_INT_ and their associated reliabilities computed from pedigree-based or single-step international beef cattle evaluations into national evaluations to obtain “blended” EBV. The Italian (ITA) pedigree-based national evaluation was used as a case study to validate the integration procedure.

**Methods:**

Publishable sires’ international information, i.e. EBV_INT_ and their associated reliabilities, was included in the national evaluation as pseudo-records. Data were available for 444,199 individual age-adjusted weaning weights of Limousin cattle from eight countries and 17,607 genotypes from four countries (ITA excluded). To mimic differences between international and national evaluations, international evaluations included phenotypes (and genotypes) of animals born prior to January 2019, while national evaluations included ITA phenotypes of animals born until April 2019. International evaluations using all available information were considered as reference scenarios. Publishable sires were divided into three groups: sires with ≥ 15, < 15 and no recorded offspring in ITA.

**Results:**

Overall, for these three groups, integrating either pedigree-based or single-step international information into national pedigree-based evaluations improved the similarity of the blended EBV with the reference EBV compared to national evaluations without integration. For instance, the correlation with the reference EBV for direct (maternal) EBV went from 0.61 (0.79) for a national evaluation without integration to 0.97 (0.88) when integrating single-step international information, on average across all groups of publishable sires.

**Conclusions:**

Our proposed one-animal-at-a-time integration procedure yields blended EBV that are in close agreement with full international EBV for all groups of animals analysed. The procedure can be directly applied by countries since it does not rely on specific software and is computationally inexpensive, allowing straightforward integration of publishable sires’ EBV_INT_ from pedigree-based or single-step based international beef cattle evaluations into national evaluations.

**Supplementary Information:**

The online version contains supplementary material available at 10.1186/s12711-023-00813-2.

## Background

International evaluations allow the comparison of estimated breeding values (EBV) across countries such that breeders can choose from a larger panel of elite bulls that better meet their selection objectives [1, 2]. Moreover, by considering relatives that are recorded in other countries, international evaluations increase the accuracy of bulls’ EBV [2–5] and reduce the potential bias of national EBV for foreign bulls [6]. In the beef cattle international evaluations that are led by Interbeef [7], national phenotypic and pedigree data from participating countries are analysed simultaneously in a multi-trait animal model in which data from each country are modelled as a separate trait [8, 9]. The main output of international evaluations is an international EBV (EBV_INT_), which usually has a higher reliability (REL) than national EBV (EBV_NAT_) [1, 4]. In Interbeef, EBV_INT_ are officially distributed to each participating country on their corresponding country scale for: (1) all the animals that appear in the national pedigree, and (2) the “publishable sires﻿”, i.e. sires that meet Interbeef publication rules (based on EBV_INT_ reliabilities and the number of recorded (grand-)progeny [10]). Thus, an individual could have two breeding values at the country level: the EBV_INT_, and the EBV_NAT_ computed from a national evaluation.

The EBV_INT_ and EBV_NAT_ can differ due to differences between national and international evaluations. For example, on the one hand, international evaluations consider information from relatives recorded in other countries but are performed within-breed and for one trait group at a time (e.g. weaning weight [1] or calving traits [11]). On the other hand, national evaluations are mostly multi-trait, can be multi-breed with data of crossbreds included, and usually include more data than those submitted for the international evaluations. One additional reason for having more data included in some national evaluations is that they usually take place according to a country-specific calendar such that national evaluations can include more recent national data compared to international evaluations.

Since national and international evaluations use partly different sources of information, choosing either the EBV_INT_ or the EBV_NAT_ for an individual can result in losing the information associated with the discarded EBV. To overcome this issue and use all available information, an integration procedure can be applied to integrate the EBV_INT_ and its associated measure of precision (e.g. its REL) into the national evaluation, resulting in a “blended” EBV [12]. An EBV_INT_ and its associated REL can be integrated as a pseudo-phenotype (e.g. de-regressed proof (DRP)) and be weighted by its associated effective record contribution (ERC) into a national evaluation. This procedure allows for the propagation of the international information to all the animals and data included in the national evaluation, as well as those excluded from the international evaluation in the first place [13]. When blending EBV_INT_ and EBV_NAT_, national information needs to be removed to avoid double-counting, which otherwise may bias national evaluations [14].

To our knowledge, an official generalized integration procedure for integrating beef cattle publishable sires’ EBV_INT_ into national evaluations is currently lacking. In dairy cattle, integration of pedigree-based and genomic-based EBV_INT_ (e.g. from multiple across-country evaluation (MACE) [15] or InterGenomics [16] international evaluations, respectively) into national evaluations is common practice [13, 14, 17, 18]. For instance, pedigree-based EBV_INT_ are integrated into national evaluations to increase the size of the national training population for genomic predictions, e.g. [17, 18]. Nonetheless, beef cattle international evaluations differ from those of dairy cattle. First, national phenotypes are directly used as input in the beef cattle international evaluation rather than using EBV as in dairy cattle international evaluations. Second, the structure of beef cattle national breeding programs usually differs from that of dairy cattle, e.g. lower usage of artificial insemination and smaller family sizes in beef compared to dairy cattle [19]. However, little research has been published on integrating EBV_INT_ at the national level for beef cattle. Pabiou et al. [20] initially tested a procedure to integrate Interbeef pedigree-based international evaluations into the Irish national evaluations. However, to date, in beef cattle, no study has investigated the integration into national evaluations of genomic EBV_INT_. Moreover, Pabiou et al. [20] used algorithms to approximate EBV and REL into DRP and ERC, which are implemented only in some commercial software packages and may not be available at the national level, potentially limiting the application of the integration procedure by countries participating in international evaluations. Thus, further testing and generalization of the integration procedure is needed to make the procedure applicable for all participating countries without relying on specific software packages, and to allow the integration of genomic EBV_INT_ from single-step international evaluations [21].

Thus, the objectives of our study were to define and validate a procedure that enables participating countries to integrate publishable sires’ international EBV that are computed using either a pedigree-based or a single-step international evaluation, into a national evaluation to obtain a blended EBV. We used data for weaning weight of Limousin cattle from countries participating in Interbeef evaluations and the Italian national dataset as a case study to validate the adequacy of the integration procedure and the predictivity of the resulting blended EBV.

## Methods

### Phenotypes, genotypes and pedigree data

Individual phenotypes for age-adjusted weaning weights (AWW) were available for 446,493 Limousin males and females. Phenotypes were available from six populations, representing eight European countries joining the Interbeef evaluations: Czech Republic (CZE), Denmark, Finland and Sweden (DFS, modelled as one population), Ireland (IRL), Germany (DEU), Switzerland (CHE), and Italy (ITA). Hereafter, for simplicity, we will refer to populations as “countries” although the DFS population is composed of data from three countries. Phenotypes from ITA came from the February 2020 Interbeef pilot evaluation, while phenotypes from the other countries came from the January 2020 Interbeef routine evaluation. Phenotypes above or below three standard deviations from the phenotypic mean of each country-sex combination were identified as outliers and discarded. After these edits, 444,199 AWW records remained, which were distributed across 20,559 herds with animals born between 1975 and 2019. DEU represented the largest country with 26% of the observations, followed by ITA (25%), DFS (22%), IRL (15%), CHE (8%), and CZE (3%). The number of phenotypes available for each country is in Table 1. Additional file 1: Table S1 shows a summary of the phenotypic distribution per country and sex. In total,17,607 genotypes (8539 males and 9068 females) imputed at a density of 57,899 single nucleotide polymorphisms (SNPs) were available and sent by four countries (Table 1). For a description of the genotypes’ preparation, imputation, and distribution per birth year, see Bonifazi et al. [21]. Hereafter, for simplicity, we will refer to phenotypes from Italy as “national” and to phenotypes and genotypes sent by other countries as “foreign”.

**Table 1:** Distribution of age-adjusted weaning weights (AWW), number of herds, year of birth of recoded animals, number and sex of genotyped animals, number of genotypes with associated phenotype for AWW in each country, and number of genotypes associated with publishable sires^b^ for direct and maternal international EBV

Country	AWW	Herds	Year of birth (min–max)	Genotypes	Genotyped males	Genotyped females	Genotypes with phenotypes^a^	Genotypes associated with publishable sires for direct (maternal) EBV
CZE	13,892	172	1991–2019	1625	730	895	1207	11 (0)
DFS	96,671	9548	1980–2019	–	–	–	–	–
IRL	68,086	8218	1975–2019	11,300	3498	7802	5,237	59 (21)
DEU	117,249	866	1981–2019	742	571	171	640^c^	216 (90)
CHE	35,695	247	1992–2018	3940	3740	200	3516	279 (56)
ITA	112,606	1508	1990–2019	–	–	–	–	–
Total	444,199	20,559	1975–2019	17,607	8539	9068	10,600	565 (167)

CZE = Czech Republic, DFS = Denmark, Finland and Sweden, IRL = Ireland, DEU = Germany, CHE = Switzerland, ITA = Italy

^a^Genotypes with an associated phenotype in the corresponding country

^b^Sires that meet Interbeef publication rules

^c^50 animals with phenotypes in DEU with genotypes sent from CHE (49) and IRL (1)

Pedigree information was extracted from the Interbeef international database. The following edits were performed: animals for which there is a pedigree loop (i.e. an animal being its ancestor), duplicated animals, and animals showing conflicts between the sex reported in the international identification number and the animal’s sex as a parent (e.g. a female reported in the pedigree as a sire) were removed. Finally, the pedigree was pruned using the RelaX2 software v1.73 [22] to include animals with phenotypes, genotypes, or both, and all their ancestors, without any limit on the number of generations retained. The final pedigree included 683,317 animals, born between 1927 and 2019, with a maximum depth of 18 generations.

### Models

#### Pedigree-based international evaluations

Pedigree-based international evaluations were implemented using the AMACI model (Animal Model accounting for Across-Country Interaction) [8] currently used in Interbeef. The AMACI model is equivalent to a multi-trait animal model with maternal effects in which each country is modelled as a different correlated trait. The international model follows the national models (Additional file 1: Table S2 reports the fixed and random effects for each country). The across-country genetic (co)variance matrix with additive direct and maternal genetic effects ($\mathbf{G}$) was built following the Interbeef procedure outlined in Bonifazi et al. [10] as $\mathbf{G}=\mathbf{S}{\varvec{\Phi}}\mathbf{S}$, where, $\mathbf{S}$ is the diagonal matrix with national genetic standard deviations for direct and maternal genetic effects, and ${\varvec{\Phi}}$ is the across-country estimated genetic correlation matrix (of order 12 × 12 with diagonal values of 1). The genetic correlation matrix ${\varvec{\Phi}}$ was estimated using the Monte Carlo expectation maximization restricted maximum likelihood (MC EM REML) algorithm implemented in the MiX99 software [23] and following the method and settings used in Bonifazi et al. [9] (“scenario ALL”). Both the estimated ${\varvec{\Phi}}$ and the final $\mathbf{G}$ (co)variance matrix are reported in Additional file 1: Table S3. Both the genetic and environmental variances were the same as those used in the national genetic evaluations of participating countries and are reported in Additional file 1: Table S4.

#### Single-step international evaluations

Genomic data were integrated into the AMACI model using the international single-step single nucleotide polymorphism best linear unbiased prediction (ssSNPBLUP) model following Bonifazi et al. [21]. The estimated (co)variance components used in ssSNPBLUP were the same as in the AMACI model. The proportion of variance not explained by SNPs and due to residual polygenic effects was assumed to be 5%. Two $\mathbf{J}$ covariates (one for the additive genetic effect and one for the maternal genetic effect) were fitted to ensure the compatibility of the pedigree and genomic information [24]. For more details on how $\mathbf{J}$ covariates are calculated see Bonifazi et al. [21].

#### National evaluations

National evaluations for ITA were always pedigree-based as no genomic data were sent by ITA. National evaluations were obtained by running a single-trait evaluation using only the phenotypes submitted by ITA and the same national model as that used for the international evaluations.

#### Reliabilities

All reliabilities were computed using the MiXBLUP software [25] and were expressed on a 0 to 1 scale. For pedigree-based national and international evaluations, REL were computed using the algorithm of Tier and Meyer [26]. Since there is no method to easily approximate REL from ssSNPBLUP models, REL were obtained from an equivalent ssGBLUP model [27, 28] using a 5% residual polygenic effect. When the same parametrization is used, ssGBLUP and ssSNPBLUP are equivalent [29]. For single-step international evaluations, the additional REL brought by genomic data was computed using the “approx1” algorithm of Misztal et al. [30] without propagation of genomic information to non-genotyped animals.

### Integration procedure

**Fig. 1:**
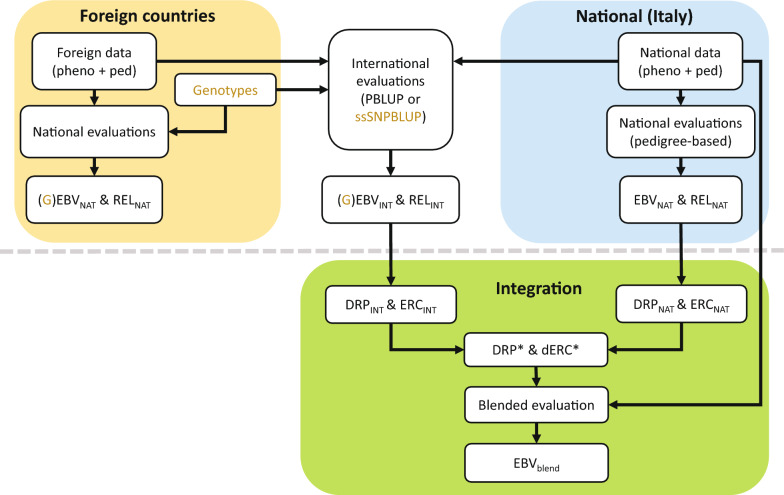
Data exchange in the Interbeef international evaluations (above the dotted line) and integration procedure (green area). Italy and foreign countries each run independent national evaluations using nationally available information: pedigree (ped), phenotypes (pheno) and genotypes (in yellow). National phenotypic and pedigree information are used in pedigree-based international evaluations to compute international EBV (EBV_INT_). If available, genotypes can be used in ssSNPBLUP international evaluations (in yellow) to compute international genomic EBV (GEBV_INT_). ssSNPBLUP evaluations are not yet part of routine Interbeef evaluations. *PBLUP* pedigree-based BLUP, *ssSNPBLUP* single-step SNPBLUP, *(G)EBV* (genomic) estimated breeding value, *REL* reliability, *INT* international, *NAT* national, *EBV*_*blend*_ blended EBV, *DRP* de-regressed proofs, *ERC* effective record contribution, DRP* adjusted DRP, dERC* adjusted de-regressed ERC

Figure 1 summarizes the exchange of data in the Interbeef international evaluations and the steps of the integration procedure outlined hereafter. The direct and maternal EBV from national (EBV_NAT_) and international (EBV_INT_) evaluations and their associated reliabilities (REL_NAT_ and REL_INT_, respectively) for all individuals in the evaluations are computed following the models outlined above. The procedure to integrate international information (i.e. EBV_INT_ and associated REL_INT_) (either pedigree-based or from single-step) into national evaluations comprises the following four steps:

(1) For all publishable sires, direct and maternal ERC associated with REL_NAT_ and REL_INT_ (ERC_NAT_ and ERC_INT_, respectively) are computed as:

$$ER{C}_{i}=\lambda \frac{RE{L}_{i}}{1-RE{L}_{i}},$$

where $RE{L}_{i}$ is the REL of individual $i$ (either REL_NAT_ or REL_INT_), and $\lambda ={\sigma }_{e}^{2}/{\sigma }_{a}^{2}$ with ${\sigma }_{e}^{2}$ being the national residual variance and ${\sigma }_{a}^{2}$ being either the national direct or maternal genetic variance for the direct and maternal EBV, respectively. The same $\lambda$ were used when computing ERC_NAT_ and ERC_INT_ since national variances are used in the international model. 

(2) For all publishable sires, direct and maternal DRP for both national and international EBV (DRP_NAT_ and DRP_INT_, respectively) are computed following Garrick et al. [31]:

$$DR{P}_{i}=P{A}_{i}+\frac{\left(EB{V}_{i}-P{A}_{i}\right)}{RE{{L}_{i}}_{(o+p)}},$$

where $P{A}_{i}$ is the parent average EBV of individual $i$ computed as $(EB{V}_{sire}+EB{V}_{dam})/2$, and $RE{{L}_{i}}_{(o+p)}$ is the reliability due to the individual’s own performance and of its progeny computed as ${dERC}_{i}/({dERC}_{i}+\uplambda )$. ${dERC}_{i}$ is the individual de-regressed ERC computed as $ER{C}_{i}-{ERC}_{PA}$, with ${ERC}_{PA}$ being the ERC calculated from the parent average reliability defined as ${(REL}_{sire}+{REL}_{dam})/4$. If either the national or the international $dER{C}_{i}$ is ≤ 0, both the $dER{C}_{i}$ and its associated $DR{P}_{i}$ are set to 0.

(3) For all publishable sires, the direct and maternal adjusted DRP (DRP*) and its associated weight (dERC*) adjusted for national data to avoid double-counting of national information are computed following Vandenplas et al. [14] as:

$${DRP}_{i}^{*}=\frac{{(dERC}_{IN{T}_{i}}\cdot {{DRP}_{INT}}_{i})-{(dERC}_{NA{T}_{i}}\cdot {DRP}_{NA{T}_{i}}) }{dER{C}_{i}^{*}},$$

where ${dERC}_{i}^{*}= {dERC}_{IN{T}_{i}}- {dERC}_{NA{T}_{i}}$. If ${dERC}_{i}^{*}$ is ≤ 0 or if the gain in reliability (defined as the difference between REL_INT_ and REL_NAT_) is smaller than 0.01, both $dER{C}_{i}^{*}$ and its associated ${DRP}_{i}^{*}$ are set to 0.

(4) The direct and maternal blended EBV (EBV_BLEND_) are then computed with a national evaluation using national phenotypes and direct and maternal DRP* as pseudo-phenotypes. In this blended evaluation, the animal’s direct and maternal DRP* are modelled as two additional records for the analysed trait (i.e. AWW in our study), similarly to considering them as repeated records. dERC* are used as weights for the DRP*, and the maternal DRP* are associated with the maternal effect of the animal itself and not of its dam. Two general means for the DRP* are fitted: one for the direct DRP* and one for the maternal DRP*. These means are specific to the DRP* and different from the general mean of the model. No other effects are fitted for the DRP*.

### Scenarios

The integration procedure was applied on a real-case scenario with Interbeef publishable sires’ international information integrated into the Italian evaluation. Italian evaluations are performed by ANACLI (“Associazione Nazionale Allevatori delle razze bovine Charolaise e Limousine Italiane” [32]) and currently take place in January, April, August–September, and December. Interbeef evaluations currently take place in January and October. To mimic differences between these evaluations’ calendars, Italian national evaluations were assumed to be four months later than the international evaluations, which resulted in a larger number of national phenotypes at the national level. Therefore, we integrated publishable sires’ international information from an Interbeef January 2019 evaluation into the ITA national evaluation of April 2019. Phenotypes and genotypes of animals born after April 30 2019 were discarded. We used the animal’s year of birth to include or exclude phenotypes in different scenarios since the animal weighing date for AWW was not available. Publishable sires’ international information with and without including genomic data in the international evaluations were integrated into the pedigree-based ITA evaluation. In both cases, the following scenarios were implemented to perform the integration. Table 2 summarizes the different sources of information and the purpose of each scenario. The first two scenarios implemented are needed as inputs during the integration procedure and are as follows.

**Table 2:** Overview of the implemented scenarios: names, data and purpose^a^

Scenario	Scenario type	Data^a,b^			Purpose
		Origin	Prior to January 2019	January 2019 to April 2019	
NAT_JAN_	Input	National	⬤		Used to avoid double-counting in BLEND_APR_
		Foreign			
INT_JAN_	Input	National	⬤		Publishable sires’ information to be integrated into BLEND_APR_
		Foreign	⬤		
NAT_APR_	Validation	National	⬤	⬤	A national evaluation without integration. Used for comparison with BLEND_APR_
		Foreign			
BLEND_APR_	Validation	National	⬤	⬤	A blended national evaluation that integrates publishable sires’ information of INT_JAN_ (corrected for NAT_JAN_) into NAT_APR_
		Foreign	⬤		
REF_APR_trunc_	Reference	National	⬤	⬤	Reference scenario to validate scenarios’ adequacy
		Foreign	⬤		
REF_APR_	Reference	National	⬤	⬤	Reference scenario to validate scenarios’ predictivity
		Foreign	⬤	⬤	

National = Italian phenotypes; Foreign = phenotypes and genotypes from other countries except Italy; ⬤ = data used in the scenario.

^a^The integration procedure was tested with or without genotypes in international evaluations; national evaluations were always pedigree-based

Input = scenarios the output of which is used as input in validated scenarios; Validation = scenarios validated and compared; and Reference = scenarios used as reference

^b^All scenarios used the full international pedigree

#### Scenario NAT_JAN_

A national Italian evaluation that uses only national phenotypes of animals born prior to January 2019. The purpose of this scenario is to obtain national information (i.e. EBV_NAT_ and their associated REL_NAT_) included in the international evaluation to avoid double-counting during the integration procedure.

#### Scenario INT_JAN_

An international evaluation that uses both national and foreign phenotypes (and genotypes for single-step evaluation) of animals born prior to January 2019. From this scenario, publishable sires and their international information for the integration are obtained. Publishable sires were selected separately for direct and maternal EBV_INT_ based on Interbeef publication rules as follows. Sire’s direct EBV_INT_ should have: (1) a REL_INT_ ≥ 0.5 on at least one country scale, and (2) at least 25 recorded progeny across all countries. Sire’s maternal EBV_INT_ should have: (1) an accompanying publishable direct EBV_INT_, (2) an associated REL_INT_ ≥ 0.3 on at least one country scale, and (3) at least 15 daughters with recorded progeny and at least 25 recorded grand-progeny from daughters across all countries. The total number of publishable sires was 4946 and 1707 for direct and maternal EBV_INT_, respectively. The number of publishable sires was the same regardless of whether INT_JAN_ used a pedigree-based or a single-step international evaluation. The number of genotyped publishable sires was 565 and 167, for direct and maternal EBV_INT_, respectively (Table 1).

T﻿he next two scenarios implemented are a national evaluation without integration and a national blended evaluation with integration, and are defined as follows.

#### Scenario NAT_APR_

This scenario is the same as NAT_JAN_ but uses national phenotypes of animals born until April 2019. This scenario represents a national evaluation without integration and it is used for comparison with BLEND_APR_.

#### Scenario BLEND_APR_

A blended national evaluation that uses national phenotypes as in NAT_APR_ and integrates information of publishable sires from scenario INT_JAN_ following the procedure that is described in the above section. We observed that few publishable sires (1 and 36 for direct and maternal EBV, respectively) had a dERC* = 0 in INT_JAN_ when using a single-step evaluation but had dERC* > 0 when using a pedigree-based evaluation. These differences were related to higher ERC_PA_ values when using a single-step evaluation compared to a pedigree-based evaluation. The dERC* of these few publishable sires were set to 0 in INT_JAN_ when using a pedigree-based evaluation.

The scenarios implemented up to this point mimic what would be observed and needed in real-case applications. Finally, we implemented the following two scenarios (also summarised in Table 2) with the purpose of validating different aspects of the integration procedure as described in the “Validation” section below. These scenarios are two international evaluations using various levels of phenotypes, pedigree and possibly genotype data of all involved countries.

#### Scenario REF_APR_trunc_

An international evaluation that uses national phenotypes of animals born until April 2019, and foreign phenotypes and genotypes of animals born prior to January 2019. REF_APR_trunc_ is used as a reference scenario to validate the adequacy of the integration procedure as described below.

#### Scenario REF_APR_

An international evaluation that uses both national and foreign phenotypes and genotypes of animals born until April 2019. REF_APR_ is used as a reference scenario to validate the increase in predictivity due to the integration procedure as described below.

In all implemented scenarios, the full international pedigree was used. Additional file 1: Table S5 reports the number of phenotypes and genotypes of animals born prior to January 2019 and between January 2019 and April 2019 for each country.

### Validation

We validated the integration procedure for its adequacy and for the increase in predictivity as described below by regressing the EBV of the reference scenarios (i.e. REF_APR_trunc_ and REF_APR_) on the EBV of two validation scenarios (Table 2): NAT_APR_, and BLEND_APR_. We computed the following validation metrics: Pearson’s correlation between EBV (ρ), level bias (LB – defined as the difference between the mean EBV of the validated scenario and the mean EBV of the REF scenario, and expressed in genetic standard deviations), slope (b_1_), adjusted coefficient of determination (R^2^_adj_), and root mean square error (RMSE, expressed in genetic standard deviations).

#### Adequacy

To evaluate the adequacy of the integration procedure, EBV of publishable sires from the validated scenarios were compared with the EBV obtained under the REF_APR_trunc_ scenario. REF_APR_trunc_ uses the same sources of information as in BLEND_APR_, but without approximating raw foreign phenotypic (and genomic) information into DRP and ERC. Thus, the more accurate is the integration procedure, the closer will the EBV be to those of REF_APR_trunc_. Publishable sires were divided into three groups based on having or not recorded offspring in ITA (hereafter referred to as “domestic” and “foreign” publishable sires, respectively), and the amount of recorded offspring in ITA prior to January 2019. The three groups defined were: (A) domestic publishable sires with at least 15 recorded offspring in ITA, (B) domestic publishable sires with less than 15 recorded offspring in ITA, and (C) foreign publishable sires with no recorded offspring in ITA. The number of sires with publishable direct EBV in groups A, B and C were 1382, 94 and 3470, respectively, and among these, 24, 29, and 512 were genotyped, respectively. The number of sires with publishable maternal EBV in groups A, B and C were 491, 51 and 1165, respectively, and among these, 16, 9, and 142 were genotyped, respectively.

#### Predictivity

Predictivity is defined as the ability to predict an individual’s future EBV before data (phenotypes and/or genotypes) on the animal itself or its relatives become available. For maternally-affected traits such as AWW, newly recorded individuals’ phenotype are expected to contribute to the direct EBV of their sires and to the maternal EBV of their maternal grand-sires (MGS) which express their maternal genetic effects through their daughters. Thus, to evaluate the increase in predictivity for direct EBV due to the integration procedure, EBV of the recorded offspring of publishable sires born between January 2019 and April 2019 and with records in ITA from the validated scenarios were compared with those of REF_APR_, which included four additional months (from January to April 2019) of foreign data. Offspring of publishable sires were divided into two groups: recorded offspring of publishable sires with only direct EBV_INT_ integrated (n = 1016, among which 973, 43, and 0 were offspring of sires in group A, B, and C, respectively) and recorded offspring of publishable sires with both direct and maternal EBV_INT_ integrated (n = 60, among which 53, 3 and 3 were offspring of sires in group A, B, and C, respectively). To evaluate the increase in predictivity for maternal EBV due to the integration procedure, the EBV of MGS’s daughters having recorded offspring in ITA born between January 2019 and April 2019 were compared between the validated scenarios and REF_APR_. Such MGS were publishable sires with both direct and maternal EBV integrated. In total, 740 daughters were evaluated (among which 727, 9, and 4 were daughters of sires in group A, B, and C, respectively).

Domestic sires with at least 15 recorded offspring at the national level are expected to have reliable EBV_NAT_ with small changes in their EBV_NAT_ when integrating international information. However, the effect of double-counting of national information is expected to be stronger in this group of sires compared to the others. Domestic sires with less than 15 recorded offspring are expected to have changes in their EBV_NAT_ and to benefit from the integration of international information from relatives recorded in other countries as only a few recorded offspring are available at the national level. Moreover, in this study, all domestic sires with less than 15 recorded offspring had also recorded offspring in other countries. Finally, foreign sires are expected to show the largest differences between EBV_INT_ and EBV_NAT_ as little to no information is present at the national level.

To gain insights on the level of connectedness between ITA and other countries, we also quantified the number of sires and dams with recorded offspring in ITA, followed by the number of common bulls (CB—sires with recorded offspring in ITA and other countries), and common maternal grand-sires (CMGS—maternal grand-sires with recorded grand-offspring in ITA and other countries). For each of these groups, we also quantified the number of genotyped animals provided by other countries that were present in the Italian pseudo-national pedigree to evaluate the potential increase in connectedness due to genomic data. The pseudo-national pedigree was obtained by pruning the international pedigree to include all animals with ITA phenotypes and all their ancestors.

### Software and settings

In all the scenarios, both EBV and their corresponding approximated REL were computed using the MiXBLUP software [25]. The convergence criterion of the preconditioned conjugate gradient (PCG) algorithm for the mixed model equation solutions was defined as the square root of the relative difference between solutions of two consecutive PCG iterations, and iteration was stopped when this dropped below 10^−5^. For the ssSNPBLUP models, convergence was also monitored for the CR, CK and CM criteria as defined in Vandenplas et al. [29]. Finally, custom R [33] functions were used to compute ERC, DRP, dERC* and DRP* and are available in Additional file 2.

## Results

In total, 4307 sires and 43,321 dams had recorded offspring in ITA. The average number of recorded offspring was 27.9 and 2.6 for sires and dams, respectively. In total, 217 sires had at least 100 recorded offspring. Although ITA sent no genotypes, 116 sires and three dams in the Italian pseudo-national pedigree had an associated genotype that was provided by other countries. Of these 116 sires with genotype, 76 also had recorded offspring in ITA. In total, 4453 MGS had recorded grand-offspring in ITA. Of these MGS, 574 were publishable sires for both direct and maternal EBV, 27 of which were genotyped. In total, 513 CB and 955 CMGS had recorded offspring in two or more countries. Table 3 reports the numbers of CB and CMGS connecting ITA with any other country. On average across pairs of countries, there were 122 CB and 192 CMGS. On average across pairs of countries, 44 CB and 24 CMGS were genotyped, with most of the genotypes provided by IRL and CHE (Table 3).

**Table 3:** Number (n) of (genotyped) common bulls (CB) and (genotyped) common maternal grand-sires (CMGS) connecting Italy with other countries, and country sending the genotype^a^

Country	CB		Country sending genotype^a^				CMGS		Country sending genotype^a^		
	n	With genotype	CZE	IRL	DEU	CHE	n	With genotype	IRL	DEU	CHE
CZE	101	39	1	24	2	12	192	21	17	1	3
DFS	128	38	0	24	1	13	152	20	17	0	3
IRL	132	56	0	40	2	14	171	24	20	1	3
DEU	174	53	0	35	2	16	261	31	22	3	6
CHE	74	32	0	10	1	21	182	24	10	1	13

CZE = Czech Republic, DFS = Denmark, Finland and Sweden, IRL = Ireland, DEU = Germany, CHE = Switzerland

^a^Country sending the genotype for CB or CMGS

### Publishable sires

**Fig. 2:**
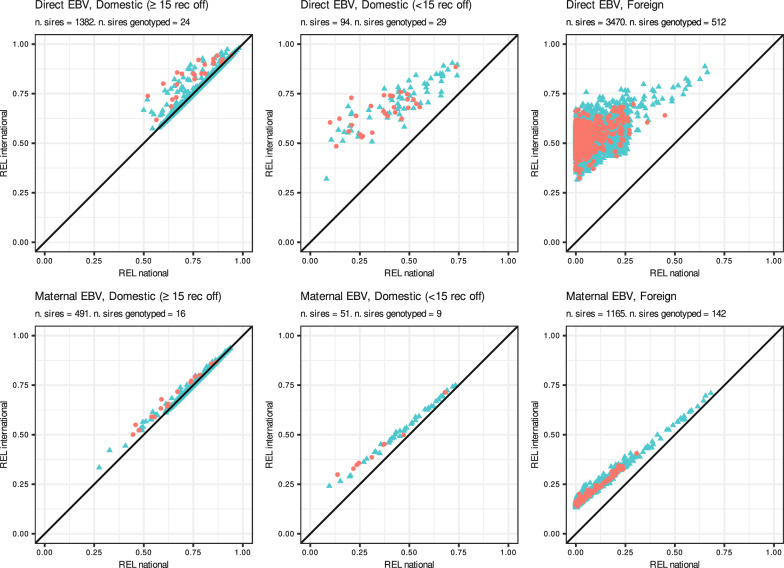
Direct EBV (top row) and maternal EBV (bottom row) reliabilities (REL) per group of publishable sires obtained from the national January evaluation (x-axis) versus the international January pedigree-based evaluation (y-axis). Red dots indicate genotyped sires. Publishable sires group: Domestic (≥ 15 rec off) correspond to publishable sires with at least 15 recorded offspring in Italy, Domestic (< 15 rec off) correspond to publishable sires with less than 15 recorded offspring in Italy, and Foreign correspond to publishable sires with no recorded offspring in Italy

The comparison of REL_INT_ from pedigree-based international evaluations to REL_NAT_ for the three groups of publishable sires shows the increase in REL obtained from international evaluations (Fig. 2). Domestic sires with at least 15 recorded offspring in ITA were associated with REL_NAT_ ≥ 0.50 for direct EBV and REL_NAT_ ≥ 0.27 for maternal EBV. In this group of sires, the pedigree-based international evaluation provided almost no increase in REL for direct EBV (0.01 points on average) and no increase in REL for the maternal EBV on average. As expected, compared to the group of sires with at least 15 recorded offspring in ITA, publishable sires with less than 15 recorded offspring in ITA were associated with lower REL_NAT_, and obtained an average increase in REL from the pedigree-based international evaluation of 0.27 points for direct EBV and of 0.06 points for maternal EBV. Finally, for both direct and maternal EBV, foreign publishable sires showed the lowest REL_NAT_ among the three groups and the highest increases in REL with the pedigree-based international evaluation, i.e. an average increase in REL of 0.45 points for direct EBV and of 0.14 for maternal EBV.

**Fig. 3:**
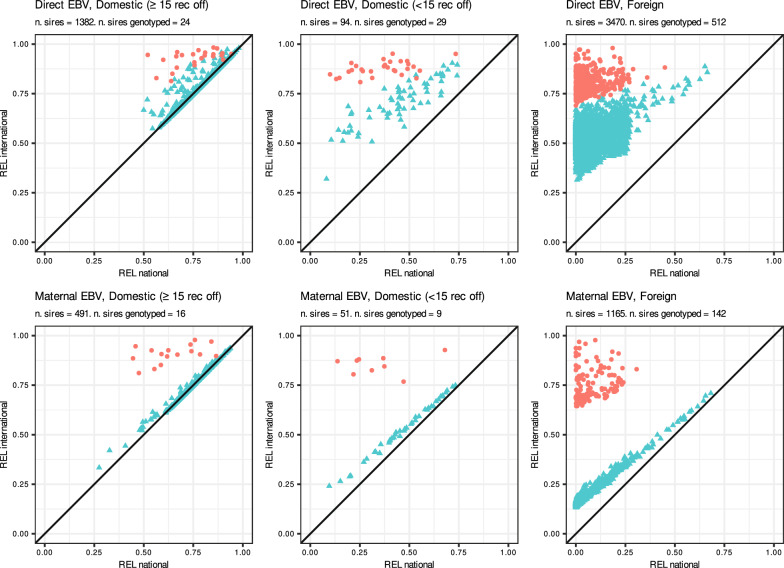
Direct EBV (top row) and maternal EBV (bottom row) reliabilities (REL) per group of publishable sires obtained from the national January evaluation (x-axis) versus the international January single-step evaluation (y-axis). Red dots indicate genotyped sires. Publishable sires group: Domestic (≥ 15 rec off) correspond to publishable sires with at least 15 recorded offspring in Italy, Domestic (< 15 rec off) correspond to publishable sires with less than 15 recorded offspring in Italy, and Foreign correspond to publishable sires with no recorded offspring in Italy

Figure 3 compares REL_INT_ from the single-step international evaluation to REL_NAT_ for the three groups of publishable sires. When a single-step international evaluation was used, for all groups of publishable sires, genotyped sires showed a higher REL_INT_ for both direct and maternal EBV compared to non-genotyped sires (Fig. 3). Non-genotyped publishable sires had the same REL_INT_ under the single-step compared to the pedigree-based international evaluations for both direct and maternal EBV (Figs. 2 and 3).

### Validation

The dERC* express in effective record contributions how much information the international evaluation adds through the integration procedure in addition to the Italian national information. The dERC* in BLEND_APR_ reflected the larger amount of international information integrated for the groups of domestic sires with less than 15 recorded offspring and foreign sires compared to that of domestic sires with at least 15 recorded offspring in ITA (see Additional file 1: Table S6). The same pattern across groups of sires was also observed when integrating information from the single-step international evaluation, but with a larger number of effective records compared to the pedigree-based international evaluation, which reflects the additional genomic information in the single-step international evaluation. On average across groups of sires, integration of information from the single-step international evaluation resulted in 2.5 and 1.3 additional effective records for direct and maternal EBV, respectively, compared to the pedigree international evaluation (see Additional file 1: Table S6).

#### Integration of pedigree-based international information into national evaluations: adequacy and predictivity

Overall, compared to NAT_APR_, BLEND_APR_ had higher ρ and R^2^_adj_, b_1_ closer to 1, LB closer to 0, and smaller RMSE (Table 4). As expected, for domestic sires with at least 15 recorded offspring, NAT_APR_ (i.e. a national evaluation without integration of international information) showed a high model adequacy for both direct and maternal EBV (ρ ≥ 0.95, b_1_ > 0.90 and R^2^_adj_ > 0.90) (Table 4). In contrast, for domestic sires with less than 15 recorded offspring and for foreign sires, the model adequacy of NAT_APR_ for both direct and maternal EBV was lower, with the group of foreign sires showing the lowest model adequacy. For direct EBV and for all groups of sires, BLEND_APR_ showed a high model adequacy (values of ρ ≥ 0.97 and b_1_ between 0.96 and 1.13) (Table 4). For maternal EBV and for both groups of domestic sires, BLEND_APR_ showed a slightly lower model adequacy compared to NAT_APR_ (difference in ρ between 0.01 and 0.02). For maternal EBV of foreign sires, BLEND_APR_ had ρ closer to 1 and b_1_ value that was even lower compared to NAT_APR_. Nonetheless, the smaller RMSE value for BLEND_APR_ suggests better model adequacy compared to NAT_APR_ (Table 4).

**Table 4:** Validation of the scenarios’ adequacy for direct and maternal EBV of publishable sires when EBV_INT_ are computed using pedigree-based international evaluations^a^

	Validation group	Scenario	ρ	LB (GSD)	b_1_	R^2^_adj_	RMSE (GSD)
Direct EBV	Domestic (≥ 15 off) (n = 1382)	NAT_APR_	0.95	− 0.14	0.96	0.90	0.18
		BLEND_APR_	0.99	0.01	0.96	0.97	0.10
	Domestic (< 15 off) (n = 94)	NAT_APR_	0.63	− 0.52	0.66	0.39	0.49
		BLEND_APR_	0.97	− 0.10	0.96	0.94	0.15
	Foreign (n = 3470)	NAT_APR_	0.24	− 0.22	0.69	0.06	0.62
		BLEND_APR_	0.97	− 0.17	1.13	0.94	0.15
Maternal EBV	Domestic (≥ 15 off) (n = 491)	NAT_APR_	0.99	0.05	1.01	0.98	0.07
		BLEND_APR_	0.98	− 0.04	0.96	0.95	0.11
	Domestic (< 15 off) (n = 51)	NAT_APR_	0.86	0.13	0.80	0.73	0.22
		BLEND_APR_	0.84	− 0.05	0.72	0.71	0.23
	Foreign (n = 1165)	NAT_APR_	0.51	0.06	0.98	0.26	0.27
		BLEND_APR_	0.83	0.06	0.57	0.69	0.17

*NAT*_*APR*_ national evaluation without integration, *BLEND*_*APR*_ blended national evaluation with integration of publishable sires’ international information and correction for double-counting

*ρ* Pearson correlation of EBV, *LB (GSD)* level bias (in genetic standard deviations), *b*_*1*_ slope, *R*^*2*^_*adj*_ adjusted R^2^, *RMSE (GSD)* root mean square error (in genetic standard deviations)

^a^The EBV of the different scenarios are compared with the pedigree-based EBV of scenario REF_APR_trunc_ (international evaluation including national data until April 2019 and foreign data prior to January 2019)

Domestic (≥ 15 off): publishable sires with at least 15 recorded offspring in Italy; Domestic (< 15 off): publishable sires with less than 15 recorded offspring in Italy; and Foreign: publishable sires with no recorded offspring in Italy

Overall, BLEND_APR_ showed a similar or higher predictivity than NAT_APR_ based on ρ, R^2^_adj_, b_1_, LB and RMSE (Table 5). NAT_APR_ showed a high predictivity for both groups of offspring of publishable sires (ρ ≥ 0.94 and b_1_ between 0.94 and 1.01) and for daughters of MGS (ρ ≥ 0.95 and b_1_ between 0.96 and 1.01) (Table 5). For direct EBV, BLEND_APR_ showed a similar or higher predictivity than NAT_APR_ for both groups of offspring of publishable sires (ρ, R^2^_adj_, and b_1_ closer to 1, LB closer to 0, and smaller RMSE) (Table 5). For maternal EBV, BLEND_APR_ showed a similar predictivity to NAT_APR_ for daughters of MGS (similar ρ, R^2^_adj_, b_1_, LB and RSME) (Table 5).

**Table 5:** Validation of the scenarios’ predictivity for direct EBV of offspring of publishable sires and for maternal EBV of daughters of MGS with publishable EBV when EBV_INT_ are computed using pedigree-based international evaluations^a^

Validation group^b^	EBV	Scenario	ρ	LB (GSD)	b_1_	R^2^_adj_	RMSE (GSD)
Offspring of sires with publishable direct EBV (n = 1016)	Direct EBV	NAT_APR_	0.96	− 0.16	0.94	0.93	0.12
		BLEND_APR_	0.99	− 0.01	0.99	0.99	0.05
	Maternal EBV	NAT_APR_	0.99	0.07	1.01	0.97	0.06
		BLEND_APR_	0.99	− 0.02	1.01	0.97	0.06
Offspring of sires with publishable direct and maternal EBV (n = 60)	Direct EBV	NAT_APR_	0.94	− 0.19	0.98	0.89	0.15
		BLEND_APR_	1.00	− 0.01	0.99	0.99	0.04
	Maternal EBV	NAT_APR_	0.99	0.07	1.01	0.98	0.05
		BLEND_APR_	0.98	− 0.04	0.94	0.96	0.07
Daughters of MGS with publishable direct and maternal EBV (n = 740)	Direct EBV	NAT_APR_	0.95	− 0.12	0.96	0.90	0.13
		BLEND_APR_	0.99	− 0.01	0.98	0.99	0.05
	Maternal EBV	NAT_APR_	0.99	0.05	1.01	0.98	0.05
		BLEND_APR_	0.99	− 0.02	0.99	0.98	0.06

*NAT*_*APR*_ national evaluation without integration, *BLEND*_*APR*_ blended national evaluation with integration of publishable sires’ international information and correction for double-counting

*ρ* Pearson correlation of EBV, *LB (GSD)* level bias (in genetic standard deviations), *b*_*1*_ slope, *R*^*2*^_*adj*_ adjusted R^2^, *RMSE (GSD)* root mean square error (in genetic standard deviations)

^a^The EBV of different scenarios are compared with pedigree-based EBV of scenario REF_APR_ (international evaluation including national data until April 2019 and foreign data until April 2019)

^b^Validation group = offspring of publishable sires for direct EBV, and for direct and maternal EBV, with records in Italy born between January 2019 and April 2019. Daughters of MGS with publishable direct and maternal EBV: maternal grand-sires’ daughters having recorded offspring in Italy born between January 2019 and April 2019

#### Integration of single-step international information into national evaluations: adequacy and predictivity

Overall, based on ρ, R^2^_adj_, b_1_, LB and RMSE, the model adequacy of BLEND_APR_ compared to NAT_APR_ was higher for direct EBV, and similar or slightly lower for maternal EBV (Table 6). The model adequacy of NAT_APR_ with the international single-step evaluation was similar to the model adequacy of NAT_APR_ with the pedigree-based international evaluation. For direct EBV and for all groups of sires, BLEND_APR_ had a higher model adequacy than NAT_APR_ (ρ ≥ 0.97 and b_1_ between 0.96 and 1.12). For maternal EBV of domestic sires with at least 15 recorded offspring and domestic sires with less than 15 recorded offspring, BLEND_APR_ had a similar or slightly lower model adequacy than NAT_APR_. For maternal EBV of foreign sires, BLEND_APR_ showed higher ρ but a b_1_ that was even lower compared to NAT_APR_; nonetheless, smaller values of RMSE and higher values of R^2^_adj_ for BLEND_APR_ suggest better model adequacy compared to NAT_APR_.

**Table 6:** Validation of the scenarios’ adequacy for direct and maternal EBV when EBV_INT_ of publishable sires are computed using single-step international evaluations^a^

	Validation group	Scenario	ρ	LB (GSD)	b_1_	R^2^_adj_	RMSE (GSD)
Direct EBV	Domestic (≥ 15 off) (n = 1382)	NAT_APR_	0.95	− 0.17	0.96	0.90	0.18
		BLEND_APR_	0.98	− 0.04	0.96	0.96	0.12
	Domestic (< 15 off) (n = 94)	NAT_APR_	0.63	− 0.55	0.66	0.39	0.49
		BLEND_APR_	0.97	− 0.20	0.97	0.93	0.16
	Foreign (n = 3470)	NAT_APR_	0.24	− 0.26	0.71	0.06	0.62
		BLEND_APR_	0.97	− 0.26	1.12	0.94	0.15
Maternal EBV	Domestic (≥ 15 off) (n = 491)	NAT_APR_	0.99	0.06	1.01	0.98	0.07
		BLEND_APR_	0.97	− 0.01	0.96	0.95	0.11
	Domestic (< 15 off) (n = 51)	NAT_APR_	0.86	0.12	0.80	0.73	0.22
		BLEND_APR_	0.85	0.00	0.72	0.71	0.23
	Foreign (n = 1165)	NAT_APR_	0.52	0.06	1.00	0.27	0.27
		BLEND_APR_	0.83	0.08	0.59	0.69	0.17

*NAT*_*APR*_ national evaluation without integration, *BLEND*_*APR*_ blended national evaluation with integration of publishable sires’ international information and correction for double-counting

*ρ* Pearson correlation of EBV, *LB (GSD)* level bias (in genetic standard deviations), *b*_*1*_ slope, *R*^*2*^_*adj*_ adjusted R^2^, *RMSE (GSD)* root mean square error (in genetic standard deviations)

^a^The EBV of different scenarios are compared with single-step EBV of scenario REF_APR_trunc_ (international evaluation including national data until April 2019 and foreign data prior to January 2019)

Domestic (≥ 15 off): publishable sires with at least 15 recorded offspring in Italy, Domestic (< 15 off): publishable sires with less than 15 recorded offspring in Italy, and Foreign: publishable sires with no recorded offspring in Italy

Overall, BLEND_APR_ showed a better predictivity compared to NAT_APR_ for direct EBV, and a similar predictivity for maternal EBV, as indicated by ρ, R^2^_adj_, b_1_, LB and RMSE (Table 7). Model predictivity of NAT_APR_ was similar to that observed for the pedigree-based international evaluation, i.e. overall, a high predictivity for both groups of offspring of publishable sires and daughters of MGS (Table 7). For direct EBV, BLEND_APR_ had a better predictivity than NAT_APR_ for both groups of offspring of publishable sires, with values of ρ, R^2^_adj_ and b_1_ closer to 1, and values of LB and RMSE closer to 0. For maternal EBV, BLEND_APR_ showed a similar predictivity to NAT_APR_ for daughters of MGS (similar ρ, R^2^_adj_, b_1_, LB and RSME) (Table 7).

**Table 7:** Validation of the scenarios’ predictivity for direct EBV of offspring of publishable sires and for maternal EBV of daughters of MGS with publishable EBV when EBV_INT_ are computed using single-step international evaluations^a^

Validation group^b^	EBV	Scenario	ρ	LB (GSD)	b_1_	R^2^_adj_	RMSE (GSD)
Offspring of sires with publishable direct EBV (n = 1016)	Direct EBV	NAT_APR_	0.96	− 0.17	0.94	0.91	0.13
		BLEND_APR_	0.99	− 0.05	1.01	0.98	0.06
	Maternal EBV	NAT_APR_	0.99	0.07	1.01	0.97	0.06
		BLEND_APR_	0.99	0.00	1.02	0.98	0.05
Offspring of sires with publishable direct and maternal EBV (n = 60)	Direct EBV	NAT_APR_	0.94	− 0.20	0.98	0.88	0.16
		BLEND_APR_	0.99	− 0.06	1.01	0.97	0.08
	Maternal EBV	NAT_APR_	0.99	0.07	1.00	0.98	0.05
		BLEND_APR_	0.98	0.00	0.94	0.95	0.08
Daughters of MGS with publishable direct and maternal EBV (n = 740)	Direct EBV	NAT_APR_	0.94	− 0.13	0.97	0.89	0.15
		BLEND_APR_	0.99	− 0.04	1.01	0.98	0.06
	Maternal EBV	NAT_APR_	0.99	0.05	1.01	0.98	0.05
		BLEND_APR_	0.99	− 0.01	0.99	0.98	0.06

*NAT*_*APR*_ national evaluation without integration, *BLEND*_*APR*_ blended national evaluation with integration of publishable sires’ international information and correction for double-counting

*ρ* Pearson correlation of EBV, *LB (GSD)* level bias (in genetic standard deviations), *b*_*1*_ slope, *R*^*2*^_*adj*_ adjusted R^2^, *RMSE (GSD)* root mean square error (in genetic standard deviations)

^a^The EBV of different scenarios are compared with single-step EBV of scenario REF_APR_ (international evaluation including national data until April 2019 and foreign data until April 2019)

^b^Validation group = offspring of publishable sires for direct EBV, and for direct and maternal EBV, with records in Italy born between January 2019 and April 2019. Daughters of MGS with publishable direct and maternal EBV: maternal grand-sires’ daughters having recorded offspring in Italy born between January 2019 and April 2019

## Discussion

National evaluations use pedigree-based or genomic-based BLUP models to estimate breeding values. A requirement for BLUP models to obtain unbiased predictions is that all the information used for selection decisions is taken into account in the current evaluation [34–36]. In practice, this requirement is not always met. For example, foreign sires that have been selected based on foreign recorded offspring may have biased national EBV since foreign records are unavailable during national evaluations [6, 37]. International evaluations allow to take the data available in other countries into account, but differences between EBV_NAT_ and EBV_INT_ may arise. In this study, we defined and validated a procedure that allows a straightforward integration of beef cattle pedigree-based and single-step EBV_INT_ into national evaluations by participating countries without relying on specific software. Hereafter, we first discuss the results of the integration procedure applied to the Italian pedigree-based national evaluations using Limousin weaning weight data, followed by a discussion on the integration procedure itself. Finally, we discuss the possible implications of this study for participating countries in the context of beef cattle international evaluations.

### Integration of pedigree-based and single-step international information

Overall, the integration of international information of publishable sires into the national pedigree-based Italian evaluation improved the model adequacy while maintaining a similar model predictivity of future international evaluations (Tables 4, 5, 6 and 7). Compared to EBV_NAT_, the blended EBV for publishable sires were in closer agreement with the international EBV of the reference scenarios. Moreover, the blended EBV showed a lower level of bias compared to EBV_NAT_. Overall, the integration procedure had greater impact for direct EBV than for maternal EBV, especially for sires with less than 15 recorded offspring and foreign sires. This was likely due to the lower REL and REL gains (REL_INT_ – REL_NAT_), which then result in smaller dERC* associated with the integrated maternal EBV_INT_ for these two groups of sires compared to either their direct EBV_INT_ and the maternal EBV_INT_ for domestic sires with at least 15 recorded offspring (Figs. 2 and 3). The lower REL of maternal EBV_INT_ compared to direct EBV_INT_ in these two groups of sires is likely due to two reasons: first, the small or null number of (grand-)offspring recorded in ITA, which provide an expression of their maternal effects; and second, the low genetic correlations between ITA and other countries for maternal effects compared to direct effects (on average 0.26 and 0.69, respectively; [see Additional file 1: Table S3]), which result in low REL of these groups of sires’ maternal EBV on the ITA scale. These results show that, due to the lower associated REL, the added benefit of the integration of publishable sires’ maternal EBV_INT_ is smaller than for direct EBV_INT_. Nonetheless, the integration procedure increased the model adequacy of national evaluations for all groups of publishable sires for both direct EBV_INT_ and maternal EBV_INT_. Finally, the integration procedure propagates the international information of publishable sires to all animals included in the national evaluation, with an impact that is proportional to the degree of relationship of the animals with the integrated sires [13]. Integrating information of publishable sires from either pedigree-based or single-step international evaluations mostly impacted the EBV of parents, and (grand-)offspring of publishable sires in the ITA evaluation (see Additional file 1 Tables S7 and S8).

The integration procedure improved pedigree-based national evaluations both when pedigree-based or single-step international information were integrated, with slightly larger improvements in model adequacy and predictivity for the former compared to the latter. Results for model adequacy are in line with those obtained by Pabiou et al. [20] who integrated pedigree-based international information into the Irish pedigree-based national evaluation. To our knowledge, our study is the first that investigates the integration of EBV_INT_ from single-step beef cattle international evaluations into pedigree-based national evaluations. The main difference between integrating single-step or pedigree-based international information is that publishable sires may have genotypes available in the international models resulting in higher REL_INT_ (Fig. 3). We further investigated possible differences in model adequacy between genotyped and non-genotyped foreign sires, since the group of foreign sires was the only one with a large number of genotyped publishable sires: 512 for direct EBV and 142 for maternal EBV (see Additional file 1: Table S9). Model adequacy was higher for foreign genotyped sires compared to non-genotyped sires for both direct and maternal EBV. This is likely due to the higher dERC* for genotyped sires which gives more weight to the international information compared to the national evaluation, resulting in blended EBV closer to the reference EBV. Genotyped publishable sires did not contribute to increasing the REL of non-genotyped sires (Figs. 2 and 3) since there was no propagation of genomic information from genotyped to non-genotyped animals. Methods to compute REL of single-step genomic evaluations as described in Liu et al. [38] could be used to propagate genomic information from genotyped to non-genotyped animals, which could potentially further improve the REL for non-genotyped publishable sires. However, such methods to compute REL in single-step genomic evaluations are still an active research topic (e.g. [39, 40]) since the approximation of REL for single-step evaluations may be computationally demanding for large datasets. Nonetheless, we expect that the propagation of genomic information from genotyped to non-genotyped animals would have little impact on the REL for non-genotyped publishable sires since they already have high associated reliabilities.

National evaluations without integration already showed high predictivity of offspring EBV (with ρ > 0.94 for direct EBV) and of daughters of MGS EBV (with ρ > 0.99 for maternal EBV) (Tables 5 and 7). The high predictivity for direct EBV of national evaluations is likely due to the offspring of publishable sires having both own phenotypes and phenotypes of national relatives (e.g. half-sibs) available at the national level, leaving little room for improvement to be made by the integration procedure. The high predictivity for maternal EBV of national evaluations for daughters of MGS is likely due to the daughters’ offspring having their own phenotypes available at the national level and to these MGS having many recorded grand-offspring at the national level (on average 83.3 per MGS), which could provide an accurate estimate of their maternal EBV. The integration procedure did not increase the predictivity further for maternal EBV mainly because these daughters were mostly from sires with at least 15 recorded offspring in ITA, for which little information was integrated on their maternal EBV (Additional file 1: Table S6). We tested whether the advantage of the integration procedure would be more pronounced when the phenotypes of offspring of publishable sires and of offspring of daughters of MGS (and that of their national and foreign contemporaries) are not yet available by integrating pedigree-based and single-step international information into NAT_JAN_ instead of NAT_APR_ (see Additional file 1: Tables S10 and S11, respectively). The integration procedure into NAT_JAN_ was performed using the integration procedure as in BLEND_APR_ (here called BLEND_JAN_). Overall, model predictivity of NAT_JAN_ was lower than that of NAT_APR,_ and the increases in predictivity due to the integration procedure were more evident, with values of ρ and b_1_ for both BLEND_JAN_ in most cases closer to 1 than those of NAT_JAN_. These results suggest that the integration procedure can increase the predictivity of national evaluations for offspring of publishable sires especially when no phenotypes are yet available on the offspring, i.e. through a more accurate parent average EBV.

### Integration procedure

Our procedure allows the integration of pedigree-based or single-step international information (EBV_INT_ and REL_INT_) into national evaluations. The proposed procedure is a simplified and generalized version of that tested by Pabiou et al. [20] in beef cattle which is similar to that proposed by Pitkänen et al. [41, 42] for dairy cattle. Compared to the methods proposed in these two studies, our procedure relies on a simplified calculation of weights (i.e. ERC) and of de-regressed EBV (i.e. DRP), using the one-animal-at-a-time formulas in steps 1 and 2 (similarly to VanRaden et al. [43]). This makes the application of the integration procedure straightforward and computationally inexpensive. More complex algorithms, such as those applied in Pabiou et al. [20] and Pitkänen et al. [41], require the availability of dedicated software packages for the computation of ERC and DRP, which may not be available at the national level. Instead, our generalized procedure can be applied by participating countries without relying on specific software. Since the beginning of international exchanges of sires, several methods to integrate different sources of information into national evaluations have been proposed [12]. However, some of these approaches, e.g. the Bayesian approaches [13, 14, 18, 44], may require adaptation of the software used for national genetic evaluations. Instead, by including external information as additional pseudo-phenotypes, the integration approach proposed in this study allows maintaining the same national model and the same software used for national routine evaluations.

In our study, we noticed that the filter for the gain in REL (defined as the difference between REL_INT_ and REL_NAT_) was key to avoid double-counting of national information for domestic sires. This filter, which is similar to that used by Pitkänen et al. [42], avoids the erroneous integration of publishable sires’ information due to approximations in REL. In particular, we noticed that such a filter improves the results for publishable sires that have no recorded offspring in other countries than ITA, by avoiding double-counting of national information. For these sires, changes in REL_INT_ compared to REL_NAT_ were due to small changes in their parent average reliability, which may be due to approximations involved in the computations of REL_INT_ and REL_NAT_. It should be noted that, in practice, the REL for a publishable sire’s EBV from routine national multi-trait evaluations may be higher than both REL_INT_ and REL_NAT_ which were computed from a single-trait evaluation in this study. Indeed, although foreign offspring records for a sire could be available for a trait evaluated in Interbeef, resulting in an associated REL_INT_ greater than the corresponding REL_NAT_, national information may be available for traits that are not yet included in Interbeef. Therefore, it is advisable to compare the REL_INT_ of publishable sires against a national REL based on the same source of information and model as the international evaluation to determine its integration. These comparable national REL have to be used as input in our integration procedure.

The scenario BLEND_APR_ avoids double-counting of national information through the adjustment of DRP and dERC in step 3 of the integration procedure. To evaluate the removal of double-counting in BLEND_APR_, we compared its results with those of a blended evaluation where double-counting of national information is avoided by integrating information from an international evaluation without national phenotypes into NAT_APR_ (GOLD scenario, [see Additional file 1: Tables S12 to S15]). The more accurate is the correction for double-counting in BLEND_APR_, the closer are the results of BLEND_APR_ expected to be to those of GOLD. Overall, dERC* in BLEND_APR_ showed good agreement with dERC* in GOLD (mean dERC* in BLEND_APR_ 0.3 effective records higher than mean dERC* in GOLD across groups of publishable sires, effects, and models; [see Additional file 1: Table S6]), indicating an appropriate removal of double-counting of national information in BLEND_APR_. Results for model adequacy and predictivity of BLEND_APR_ were close to those of GOLD. Overall, when integrating pedigree-based international information, as expected, GOLD performed slightly better than BLEND_APR_ based on model adequacy. However, BLEND_APR_ performed slightly better than GOLD for maternal EBV of foreign sires when integrating pedigree-based international information, and for both direct and maternal EBV of both domestic sires with less than 15 recorded offspring and foreign sires when integrating single-step international information. These results could be explained by the possible over-estimation of dERC* in BLEND_APR_ in comparison to dERC* in GOLD (see Additional file 1: Table S6). Step 3 of the integration procedure removes double-counting due to national records [13, 14], but this double-counting could still be present in BLEND_APR_ due to the different approximations. In GOLD, possible double-counting of national records is absent since the input international evaluation excludes national phenotypes. The effect on the blended EBV due to the remaining possible double-counting of national information in BLEND_APR_ was further investigated by regressing the blended EBV of GOLD on those of BLEND_APR_ (results not shown). Overall, when integrating pedigree-based international information, EBV correlations between GOLD and BLEND_APR_ were ≥ 0.98 for all groups of sires. When integrating single-step international information, EBV correlations between GOLD and BLEND_APR_ were equal to 0.97 for domestic sires with at least 15 recorded offspring in ITA, and ≥ 0.91 for domestic sires with less than 15 recorded offspring and foreign sires. Overall, these results suggest that the effect of double-counting of remaining national information becomes more important when integrating sires’ EBV_INT_ with lower REL compared to EBV_INT_ with high REL, in agreement with Vandenplas et al. [13]. These results also suggest that there is more double-counting when integrating single-step international information than pedigree-based information. This could be explained by the fact that genomic relationships are not considered when deregressing the international information, resulting in double-counting genomic information from the international evaluation in the blended EBV. More sophisticated and computationally demanding algorithms, such as the TSA algorithm by Vandenplas and Gengler [37], or the algorithm by Calus et al. [45] could be applied to estimate potentially more accurate weights that are free of contributions due to pedigree and genomic relationships, avoiding its double-counting and possibly further improving the results. Similarly, the de-regression step of EBV of sires could potentially be improved by using matrix de-regression procedures [31, 45–47] which, theoretically, are expected to be better than the one-animal-a-the-time de-regression proposed here [45]. However, the latter approach can be more easily applied by participating countries because it is straightforward to implement and does not rely on specific software, while it achieves sound results as shown in our study.

### Implications

Two assumptions that were applied to this study should be acknowledged for the application of the integration procedure by countries participating in Interbeef. First, the same algorithm to compute REL was used for national and international evaluations. If REL_NAT_ and REL_INT_ are approximated with different algorithms, this may cause differences between them and, in turn, differences in their corresponding ERC, which could impact the integration procedure. Thus, having an accurate and possibly the same reliability algorithm for national and international evaluations is desirable. Alternatively, when this is not possible, REL_NAT_ (or the corresponding dERC, similarly to what is done in MACE evaluations [17, 48]) could be computed and distributed at the international level after performing pseudo-national evaluations using the same reliability algorithm as that used for international evaluations. These pseudo-national evaluations can be obtained by running a pedigree-based or single-step evaluation for each country and using only national data. The second assumption was that EBV_INT_ were already expressed on the same scale as EBV_NAT_. If the EBV_INT_ or the EBV_NAT_ are expressed on different scales or genetic bases, such differences could impact the integration [31] and need to be taken into account (as in e.g. [18]) before starting the integration procedure. Systematic differences between EBV_INT_ and EBV_NAT_ can simply be accounted for by fitting a general mean or an overall fixed effect per each DRP* group, i.e., one for direct DRPs* and one for maternal DRPs*, as done in this study.

The proposed integration procedure can be applied by countries participating in Interbeef evaluations to integrate publishable sires’ EBV_INT_ at the national level. Integrating information from an international evaluation that excludes national phenotypes (as in scenario GOLD above) would be optimal because it completely avoids double-counting of national information. However, this integration requires to compute and distribute, for each country, EBV and REL from an international evaluation from which the country’s national data is removed. Instead, integrating information as in scenario BLEND_APR_ can be directly applied at the country level using information already available. Applying the integration as in BLEND_APR_ implies that a pseudo-national evaluation with the same information as provided to Interbeef should be performed to remove possible double-counting during the integration. This pseudo-national evaluation can be performed at the country level or at the international level as explained above. In the latter case, the resulting EBV_NAT_ and REL_NAT_ could be distributed next to the EBV_INT_ and REL_INT_.

Our results suggest that the integration of single-step international information is able to adequately make use of external genomic information. As ITA national evaluations were pedigree-based, no double-counting due to domestic genotypes was present when performing the integration. When integrating single-step international information into single-step national evaluations, a similar procedure as that proposed here can be used. However, double-counting of national genomic information should be removed from the international single-step evaluation prior to the integration [13]. Thus, our proposed method should be adapted to avoid double-counting of national genomic information, and further research is needed.

Finally, we expect that the integration procedure would give similar results when applied to other traits and breeds evaluated in Interbeef since similar rules for the publication of sires’ EBV_INT_ apply. The proposed integration procedure could be applied to any animal with an available EBV_INT_ (and associated REL_INT_). However, the adequacy of the integration procedure to integrate international information for animals with low associated REL (e.g. cows) is currently unknown and should be further investigated since the approximation of information into DRP could be sensitive to the low REL of EBV.

## Conclusions

We propose a general integration procedure to integrate beef cattle international EBV of publishable sires computed from either pedigree-based or single-step evaluations into national evaluations. Using weaning weight of Limousin cattle from countries participating in Interbeef evaluations and the Italian pedigree-based national evaluations as a case study, we showed that the proposed integration procedure increased the model adequacy for EBV of publishable sires, while giving a similar or higher predictivity for EBV of their domestic offspring. The procedure worked well both when integrating information either from pedigree-based international evaluations or from single-step international evaluations. The proposed one-animal-at-a-time integration procedure is computationally inexpensive and its application to existing national evaluations is straightforward since it does not require any specific software or adaptation of those used in national routine evaluations.

## Supplementary Information

**Additional file 1: Table S1.** Phenotypic distribution of AWW per country for males and females**. Table S2.** List of environmental effects in each national model. **Table S3.** Direct and maternal genetic covariances (below diagonal), genetic variances (diagonal) and genetic correlations (above diagonal) within and across countries. **Table S4.** National genetic, environmental, and residual variances. **Table S5.** Number of phenotypes and genotypes per country used in the implemented scenarios. **Table S6.** Distribution of adjusted de-regressed effective record contribution (dERC*) for direct and maternal EBV of publishable sires, from either pedigree-based or single-step international evaluations. **Table S7.** Comparison of direct and maternal EBV for all animals in the Italian pseudo-national pedigree between NAT_APR_ and BLEND_APR_ integrating EBV from pedigree-based international evaluations. **Table S8.** Comparison of direct and maternal EBV for all animals in the Italian pseudo-national pedigree between NAT_APR_ and BLEND_APR_ integrating EBV from single-step international evaluations. **Table S9.** Validation of the scenarios’ adequacy for direct and maternal EBV of genotyped and non-genotyped foreign publishable sires when EBV_INT_ are computed using single-step international evaluations. **Table S10.** Validation of the scenarios’ predictivity for direct EBV of offspring of publishable sires and for maternal EBV of daughters of MGS with publishable EBV when international information are integrated on Scenario NAT_JAN_ and EBV_INT_ are computed using pedigree-based international evaluations. **Table S11.** Validation of the scenarios’ predictivity for direct EBV of offspring of publishable sires and for maternal EBV of daughters of MGS with publishable EBV when international information are integrated on Scenario NAT_JAN_ and EBV_INT_ are computed using single-step international evaluations. **Table S12.** Validation of the GOLD scenario’s adequacy for direct and maternal EBV of publishable sires when EBV_INT_ are computed using pedigree-based international evaluations. **Table S13.** Validation of the GOLD scenario’s predictivity for direct EBV of offspring of publishable sires and for maternal EBV of daughters of MGS with publishable EBV when EBV_INT_ are computed using pedigree-based international evaluations. **Table S14.** Validation of the GOLD scenario’s adequacy for direct and maternal EBV of publishable sires when EBV_INT_ are computed using single-step international evaluations. **Table S15.** Validation of the GOLD scenario’s predictivity for direct EBV of offspring of publishable sires and for maternal EBV of daughters of MGS with publishable EBV when EBV_INT_ are computed using single-step international evaluations.

**Additional file 2.** R functions to compute ERC, DRP, dERC* and DRP* (also available at https://github.com/bonifazi/Integration_EBV_and_GEBV).

## Data Availability

All information supporting the results are included in this article and its additional files. The data that support the findings of this study are available at Interbeef. Restrictions apply to the availability of these data, which were used under license for the current study.
