# Supplementary material for: Integration of beef cattle international pedigree and genomic estimated breeding values into national evaluations, with an application to the Italian Limousin population
Source: Genet Sel Evol. 2023 Jun 12;55:41. doi: 10.1186/s12711-023-00813-2 (PMC10258954; doi:10.1186/s12711-023-00813-2)
Supplement: Supplementary file 1 — Additional file 1: Table S1. Phenotypic distribution of AWW per country for males and females. Table S2. List of environmental effects in each national model. Table S3. Direct and maternal genetic covariances (below diagonal), genetic variances (diagonal) and genetic correlations (above diagonal) within and across countries. Table S4. National genetic, environmental, and residual variances. Table S5. Number of phenotypes and genotypes per country used in the implemented scenarios. Table S6. Distribution of adjusted de-regressed effective record contribution (dERC*) for direct and maternal EBV of publishable sires, from either pedigree-based or single-step international evaluations. Table S7. Comparison of direct and maternal EBV for all animals in the Italian pseudo-national pedigree between NATAPR and BLENDAPR integrating EBV from pedigree-based international evaluations. Table S8. Comparison of direct and maternal EBV for all animals in the Italian pseudo-national pedigree between NATAPR and BLENDAPR integrating EBV from single-step international evaluations. Table S9. Validation of the scenarios’ adequacy for direct and maternal EBV of genotyped and non-genotyped foreign publishable sires when EBVINT are computed using single-step international evaluations. Table S10. Validation of the scenarios’ predictivity for direct EBV of offspring of publishable sires and for maternal EBV of daughters of MGS with publishable EBV when international information are integrated on Scenario NATJAN and EBVINT are computed using pedigree-based international evaluations. Table S11. Validation of the scenarios’ predictivity for direct EBV of offspring of publishable sires and for maternal EBV of daughters of MGS with publishable EBV when international information are integrated on Scenario NATJAN and EBVINT are computed using single-step international evaluations. Table S12. Validation of the GOLD scenario’s adequacy for direct and maternal EBV of publishable sires when EBVINT are c [file 12711_2023_813_MOESM1_ESM.docx]

# Additional file 1

Table S1. Phenotypic distribution of AWW per country for males and females ^a^.

| COU ^b^ | Males | | | | | Females | | | | |  |
| --- | --- | --- | --- | --- | --- | --- | --- | --- | --- | --- | --- |
|  | N | Min | Mean | Max | $\sigma_{P}$ | N | Min | Mean | Max | $\sigma_{P}$ | |
| CZE | 6,816 | 173 | 293.2 | 411 | 37.8 | 7,076 | 157 | 262.8 | 366 | 33.5 | |
| DFS | 48,340 | 112 | 240.6 | 369 | 41.5 | 48,331 | 107 | 213.3 | 319 | 34.1 | |
| IRL | 40,873 | 134 | 297.2 | 460 | 53.9 | 27,213 | 127 | 264.4 | 402 | 45.2 | |
| DEU | 58,716 | 137 | 269.9 | 402 | 43.3 | 58,533 | 128 | 242.3 | 356 | 37.0 | |
| CHE | 18,197 | 112 | 233.4 | 354 | 39.2 | 17,498 | 107 | 209.7 | 312 | 33.2 | |
| ITA | 49,100 | 75 | 206.1 | 339 | 42.8 | 63,506 | 75 | 196.9 | 320 | 39.8 | |

^a^ AWW = age-adjusted weaning weights, N = number of phenotypes, Min = minimum, Max = maximum, $\sigma_{P}$ = phenotypic standard deviation.

^b^ COU = Country: CZE = Czech Republic, DFS = Denmark, Finland and Sweden, IRL = Ireland, DEU = Germany, CHE = Switzerland, ITA = Italy.

Table S2. List of environmental effects in each national model ^a, b^.

| **COU ^a^** | **Fixed** | | | | | | **Random** | | **Covariates** | |  |
| --- | --- | --- | --- | --- | --- | --- | --- | --- | --- | --- | --- |
| CZE |  | asextwin |  | year |  |  | PE | HYS | aaca | aaca2 |  |
| DFS | HYS | asex | aaca | seas | twin |  | PE |  |  |  |  |
| IRL | HYS | asex | pariagedam |  |  |  | PE |  | agedam2 | aawg |  |
| DEU |  | asex | pari | month | twin |  |  | HY |  |  |  |
| CHE |  | asex |  | yearmonth |  | alpine | PE | HY | agedam | agedam2 |  |
| ITA | HYS | asex |  |  | twin |  | PE |  | aawg | aaca | aaca2 |

^a^ COU = Country: CZE = Czech Republic, DFS = Denmark, Finland and Sweden, IRL = Ireland, DEU = Germany, CHE = Switzerland, ITA = Italy.

^b^ aaca = age at calving; aaca2 = age at calving as quadratic effect; aawg = age at weighting; agedam = age of the dam; agedam2 = age of the dam fitted as quadratic effect; alpine = access to alpine grazing for calves; asex = sex of the animal; asextwin = interaction between asex and twin; HY = Herd-Year; HYS = Herd-Year-Season; month = month of birth; pari = parity; pariagedam = interaction between pari and agedam; PE = maternal permanent environmental effect; seas = season; twin = twinning; year = year of birth; yearmonth = interaction between year and month.

Table S3. Direct and maternal genetic covariances (below diagonal), genetic variances (diagonal) and genetic correlations (above diagonal) within and across countries ^a^.

|  |  | Direct | | | | | | Maternal | | | | | |
| --- | --- | --- | --- | --- | --- | --- | --- | --- | --- | --- | --- | --- | --- |
|  |  | CZE | DFS | IRL | DEU | CHE | ITA | CZE | DFS | IRL | DEU | CHE | ITA |
| Direct | CZE | 686 | 0.73 | 0.64 | 0.64 | 0.72 | 0.69 | -0.39 | -0.14 | -0.05 | -0.04 | -0.13 | -0.39 |
|  | DFS | 313.97 | 269 | 0.56 | 0.86 | 0.72 | 0.73 | 0.01 | -0.39 | -0.08 | -0.07 | -0.16 | -0.39 |
|  | IRL | 354.93 | 194.97 | 450 | 0.45 | 0.56 | 0.63 | -0.02 | -0.19 | -0.41 | -0.09 | -0.12 | -0.39 |
|  | DEU | 326.74 | 275.66 | 184.92 | 383 | 0.62 | 0.68 | -0.01 | -0.22 | -0.07 | -0.38 | -0.14 | -0.38 |
|  | CHE | 369.97 | 229.16 | 232.38 | 235.53 | 380 | 0.70 | -0.02 | -0.19 | -0.07 | -0.06 | -0.47 | -0.40 |
|  | ITA | 175.75 | 116.63 | 130.45 | 128.58 | 132.86 | 94 | 0.22 | 0.05 | 0.16 | 0.15 | 0.09 | -0.66 |
| Maternal | CZE | -144.99 | 2.37 | -5.11 | -1.95 | -4.39 | 29.98 | 197 | 0.37 | 0.42 | 0.46 | 0.48 | 0.22 |
|  | DFS | -40.89 | -69.68 | -43.09 | -46.61 | -39.94 | 5.35 | 56.75 | 120 | 0.54 | 0.56 | 0.53 | 0.31 |
|  | IRL | -19.59 | -18.23 | -119.87 | -19.09 | -19.70 | 21.29 | 82.03 | 81.80 | 194 | 0.57 | 0.49 | 0.23 |
|  | DEU | -19.02 | -21.30 | -32.76 | -132.86 | -21.20 | 27.13 | 117.18 | 110.61 | 143.68 | 326 | 0.54 | 0.25 |
|  | CHE | -33.30 | -25.32 | -25.53 | -26.50 | -89.26 | 8.86 | 65.06 | 56.00 | 66.73 | 94.08 | 94 | 0.28 |
|  | ITA | -89.19 | -56.68 | -72.20 | -66.45 | -68.20 | -56.44 | 27.05 | 29.79 | 28.84 | 40.62 | 23.69 | 78 |

^a^ Country: CZE = Czech Republic, DFS = Denmark, Finland and Sweden, IRL = Ireland, DEU = Germany, CHE = Switzerland, ITA = Italy.

Table S4. National genetic, environmental, and residual variances ^b^.

| **COU ^a^** | **σ^2^_HY_** | **σ^2^_HYS_** | **σ^2^_PE_** | **σ^2^_dir_** | **σ^2^_mat_** | **σ^2^_res_** |
| --- | --- | --- | --- | --- | --- | --- |
| CZE |  | 294 | 208 | 686 | 197 | 377 |
| DFS |  |  | 90 | 269 | 120 | 547 |
| IRL |  |  | 45 | 450 | 194 | 647 |
| DEU | 477 |  |  | 383 | 326 | 719 |
| CHE | 203 |  | 76 | 380 | 94 | 587 |
| ITA |  |  | 69 | 94 | 78 | 278 |

^a^ Country: CZE = Czech Republic, DFS = Denmark, Finland and Sweden, IRL = Ireland, DEU = Germany, CHE = Switzerland, ITA = Italy.

^b^ σ^2^ = variance, HY = Herd-Year, HYS = Herd-Year-Season, PE = maternal permanent environment, dir = direct genetic effect, mat = maternal genetic effect, res = residual.

Table S5. Number of phenotypes and genotypes per country ^a^ used in the implemented scenarios.

|  | **Phenotypes** | |  | **Genotypes** | |  |
| --- | --- | --- | --- | --- | --- | --- |
| **COU ^a^** | Prior to  January 2019 | January 2019 to April 2019 | Total | Prior to  January 2019 | January 2019 to  April 2019 | Total |
| CZE | 13,741 | 151 | 13,892 | 1,298 | 286 | 1,584 |
| DFS | 94,852 | 1,495 | 96,347 | - | - | - |
| IRL | 66,621 | 1,422 | 68,043 | 11,300 | - | 11,300 |
| DEU | 113,993 | 2,137 | 116,130 | 626 | 90 | 716 |
| CHE | 35,695 | - | 35,695 | 3,922 | 15 | 3,937 |
| ITA | 109,283 | 2,301 | 111,584 | - | - | - |
| Total | 434,185 | 7,506 | 441,691 | 17,146 | 391 | 17,537 |

^a^ COU = Country: CZE = Czech Republic, DFS = Denmark, Finland and Sweden, IRL = Ireland, DEU = Germany, CHE = Switzerland, ITA = Italy.

Table S6. Distribution of adjusted de-regressed effective record contribution (dERC*) ^a^ for direct and maternal EBV of publishable sires, from either pedigree-based or single-step international evaluations.

|  |  |  | **International evaluation** | | | | | | | |
| --- | --- | --- | --- | --- | --- | --- | --- | --- | --- | --- |
|  |  |  | **pedigree-based** | | | | **single-step** | | | |
|  | **Validation**  **group ^b^** | **Scenario ^c^** | **min** | **mean** | **max** | **n > 0** | **min** | **mean** | **max** | **n > 0** |
| Direct  EBV | Domestic (≥ 15 off)  (n = 1,382) | BLEND_APR_ | 0.0 | 0.5 | 71.1 | 97 | 0.0 | 1.0 | 152.9 | 98 |
|  |  | GOLD | 0.0 | 0.3 | 11.6 | 121 | 0.0 | 0.8 | 138.2 | 123 |
|  | Domestic (< 15 off)  (n = 94) | BLEND_APR_ | 0.9 | 5.2 | 20.3 | 94 | 0.9 | 10.5 | 55.6 | 94 |
|  |  | GOLD | 0.9 | 4.0 | 10.0 | 94 | 0.9 | 9.2 | 54.9 | 94 |
|  | Foreign  (n = 3,470) | BLEND_APR_ | 0.9 | 2.5 | 17.4 | 3,470 | 0.3 | 4.0 | 145.3 | 3,470 |
|  |  | GOLD | 0.9 | 2.4 | 9.3 | 3,470 | 0.3 | 3.9 | 144.0 | 3,470 |
| Maternal  EBV | Domestic (≥ 15 off)  (n=491) | BLEND_APR_ | 0.0 | 0.1 | 3.6 | 52 | 0.0 | 1.4 | 149.0 | 53 |
|  |  | GOLD | 0.0 | 0.1 | 1.2 | 70 | 0.0 | 1.3 | 134.5 | 74 |
|  | Domestic (< 15 off)  (n=51) | BLEND_APR_ | 0.3 | 0.7 | 1.3 | 51 | 0.3 | 4.3 | 37.9 | 51 |
|  |  | GOLD | 0.4 | 0.7 | 1.1 | 51 | 0.0 | 4.1 | 35.9 | 50 |
|  | Foreign  (n=1,165) | BLEND_APR_ | 0.0 | 0.5 | 1.3 | 1,129 | 0.0 | 2.1 | 152.2 | 1,129 |
|  |  | GOLD | 0.3 | 0.4 | 1.1 | 1,165 | 0.0 | 2.0 | 148.7 | 1,112 |

^a^ dERC* represents additional information added on top of NAT_APR_. min: minimum, max: maximum, n > 0: number of dERC* greater than 0.

^b^ Validation group = Domestic (≥ 15 off): publishable sires with at least 15 recorded offspring in Italy, Domestic (< 15 off): publishable sires with less than 15 recorded offspring in Italy, and Foreign: publishable sires with no recorded offspring in Italy.

^c^ Scenario: BLEND_APR_: blended national evaluation with integration of publishable sires’ international information and correction for double-counting, GOLD: as BLEND_APR_, but integrating publishable sires’ international information from an international evaluation that did not include national data.

Table S7. Comparison ^a^ of direct and maternal EBV for all animals in the Italian pseudo-national pedigree ^b^ between NAT_APR_ and BLEND_APR_ integrating EBV from pedigree-based international evaluations ^c^.

|  | **Group of animals ^d^** | **n animals** | **min** | **mean** | **max** | **std** |
| --- | --- | --- | --- | --- | --- | --- |
| Direct EBV | Offspring of pub. sires Domestic (≥ 15 off) | 97,507 | -2.47 | -0.08 | 0.62 | 0.13 |
|  | Offspring of pub. sires Domestic (< 15 off) | 2,352 | -1.26 | -0.13 | 0.71 | 0.35 |
|  | Offspring of pub. sires Foreign | 1,811 | -1.89 | -0.08 | 1.13 | 0.39 |
|  | Grand-offspring of pub. sires | 29,043 | -1.13 | 0.02 | 1.22 | 0.13 |
|  | Parents of pub. sires | 1,239 | -1.40 | 0.00 | 1.10 | 0.28 |
|  | Other animals | 59,837 | -1.37 | -0.01 | 0.72 | 0.14 |
| Maternal EBV | Offspring of pub. sires Domestic (≥ 15 off) | 97,507 | -0.41 | 0.05 | 1.57 | 0.08 |
|  | Offspring of pub. sires Domestic (< 15 off) | 2,352 | -0.48 | 0.08 | 0.86 | 0.23 |
|  | Offspring of pub. sires Foreign | 1,811 | -0.69 | 0.07 | 1.14 | 0.27 |
|  | Grand-offspring of pub. sires | 29,043 | -0.93 | -0.01 | 0.70 | 0.08 |
|  | Parents of pub. sires | 1,239 | -0.74 | 0.00 | 0.85 | 0.19 |
|  | Other animals | 59,837 | -0.47 | 0.01 | 0.84 | 0.10 |

^a^ Comparison show the minimum (min), mean, maximum (max), and standard deviation (std) of the difference between EBV of NAT_APR_ and EBV of BLEND_APR_. All values are expressed in genetic standard deviations units.

^b^ the pseudo-national pedigree was obtained by pruning the international pedigree to include all animals with ITA phenotypes and all their ancestors.

^c^ NAT_APR_: national evaluation without integration, BLEND_APR_: blended national evaluation with integration of publishable sires’ international information and correction for double-counting.

^d^ Groups of animals: Offspring of pub. sires Domestic (≥ 15 off) = Offspring of domestic publishable sires with at least 15 recorded offspring in Italy, Offspring of pub. sires Domestic (< 15 off) = Offspring of domestic publishable sires with less than 15 recorded offspring in Italy, Offspring of pub. sires Foreign = Offspring of domestic publishable sires with no recorded offspring in Italy, Grand-offspring of pub. Sires = Grand-offspring of publishable sires, Parents of pub. sires = Parents of publishable sires, Other animals = all other animals in the pedigree excluding publishable sires.

Table S8. Comparison ^a^ of direct and maternal EBV for all animals in the Italian pseudo-national pedigree ^b^ between NAT_APR_ and BLEND_APR_ integrating EBV from single-step international evaluations ^c^.

|  | **Group of animals ^d^** | **n animals** | **min** | **mean** | **max** | **std** |
| --- | --- | --- | --- | --- | --- | --- |
| Direct EBV | Offspring of pub. sires Domestic (≥ 15 off) | 97,507 | -2.69 | -0.07 | 0.69 | 0.12 |
|  | Offspring of pub. sires Domestic (< 15 off) | 2,352 | -1.27 | -0.10 | 0.82 | 0.34 |
|  | Offspring of pub. sires Foreign | 1,811 | -2.06 | -0.05 | 1.26 | 0.40 |
|  | Grand-offspring of pub. sires | 29,043 | -1.07 | 0.00 | 1.24 | 0.13 |
|  | Parents of pub. sires | 1,239 | -1.27 | 0.02 | 1.11 | 0.28 |
|  | Other animals | 59,837 | -1.44 | -0.01 | 0.80 | 0.15 |
| Maternal EBV | Offspring of pub. sires Domestic (≥ 15 off) | 97,507 | -0.31 | 0.04 | 1.71 | 0.08 |
|  | Offspring of pub. sires Domestic (< 15 off) | 2,352 | -0.50 | 0.05 | 0.83 | 0.22 |
|  | Offspring of pub. sires Foreign | 1,811 | -0.75 | 0.05 | 1.27 | 0.27 |
|  | Grand-offspring of pub. sires | 29,043 | -0.91 | 0.00 | 0.70 | 0.08 |
|  | Parents of pub. sires | 1,239 | -0.72 | -0.02 | 0.75 | 0.19 |
|  | Other animals | 59,837 | -0.54 | 0.00 | 0.90 | 0.09 |

^a^ Comparison show the minimum (min), mean, maximum (max), and standard deviation (std) of the difference between EBV of NAT_APR_ and EBV of BLEND_APR_. All values are expressed in genetic standard deviations units.

^b^ the pseudo-national pedigree was obtained by pruning the international pedigree to include all animals with ITA phenotypes and all their ancestors.

^c^ NAT_APR_: national evaluation without integration, BLEND_APR_: blended national evaluation with integration of publishable sires’ international information and correction for double-counting.

^d^ Groups of animals: Offspring of pub. sires Domestic (≥ 15 off) = Offspring of domestic publishable sires with at least 15 recorded offspring in Italy, Offspring of pub. sires Domestic (< 15 off) = Offspring of domestic publishable sires with less than 15 recorded offspring in Italy, Offspring of pub. sires Foreign = Offspring of domestic publishable sires with no recorded offspring in Italy, Grand-offspring of pub. Sires = Grand-offspring of publishable sires, Parents of pub. sires = Parents of publishable sires, Other animals = all other animals in the pedigree excluding publishable sires.

Table S9. Validation of the scenarios’ adequacy for direct and maternal EBV of genotyped and non-genotyped foreign publishable sires when EBV_INT_ are computed using single-step international evaluations ^a^.

|  |  |  |  |  |  |  |  | **Summary dERC*** | | | |
| --- | --- | --- | --- | --- | --- | --- | --- | --- | --- | --- | --- |
|  | **Foreign**  **sires** | **Scenario ^b^** | **ρ** | **LB**  **(GSD)** | **b_1_** | **R^2^_adj_** | **RMSE (GSD)** | **min** | **mean** | **max** | **n > 0** |
| Direct  EBV | Genotyped  (n = 512) | NAT_APR_ | 0.18 | -0.37 | 0.61 | 0.03 | 0.62 | - | - | - | - |
|  |  | BLEND_APR_ | 0.99 | -0.33 | 1.07 | 0.99 | 0.07 | 6.3 | 12.6 | 145.3 | 512 |
|  |  | GOLD | 0.94 | -0.32 | 0.99 | 0.89 | 0.21 | 6.3 | 12.5 | 144.0 | 512 |
|  | Non-genotyped  (n = 2,958) | NAT_APR_ | 0.25 | -0.25 | 0.72 | 0.06 | 0.61 | - | - | - | - |
|  |  | BLEND_APR_ | 0.97 | -0.25 | 1.13 | 0.94 | 0.16 | 0.3 | 2.5 | 17.4 | 2958 |
|  |  | GOLD | 0.90 | -0.18 | 1.05 | 0.81 | 0.28 | 0.3 | 2.4 | 9.3 | 2958 |
| Maternal  EBV | Genotyped  (n = 142) | NAT_APR_ | 0.55 | 0.07 | 1.18 | 0.30 | 0.28 | - | - | - | - |
|  |  | BLEND_APR_ | 0.97 | 0.23 | 0.88 | 0.93 | 0.09 | 6.2 | 13.6 | 152.2 | 142 |
|  |  | GOLD | 0.87 | 0.14 | 0.81 | 0.75 | 0.17 | 6.2 | 13.3 | 148.7 | 142 |
|  | Non-genotyped  (n = 1,023) | NAT_APR_ | 0.52 | 0.06 | 0.98 | 0.27 | 0.26 | - | - | - | - |
|  |  | BLEND_APR_ | 0.82 | 0.06 | 0.57 | 0.68 | 0.17 | 0.0 | 0.5 | 1.3 | 987 |
|  |  | GOLD | 0.73 | -0.02 | 0.51 | 0.54 | 0.21 | 0.0 | 0.4 | 1.1 | 970 |

^a^ The EBV of the different scenarios are compared with single-step EBV of scenario REF_APR_trunc_ (international evaluation including national data until April 2019 and foreign data prior to January 2019).

ρ: Pearson correlation of EBV, LB (GSD): level bias (in genetic standard deviations), b_1_: slope, R^2^_adj_: adjusted R^2^, RMSE (GSD): Root Mean Square Error (in genetic standard deviations), dERC*: adjusted de-regressed effective record contribution (representing additional information added on top of NAT_APR_), min: minimum, max: maximum, n > 0: number of dERC* greater than 0.

^b^ Scenario: NAT_APR_: national evaluation without integration, BLEND_APR_: blended national evaluation with integration of publishable sires’ international information and correction for double-counting, GOLD: as BLEND_APR_, but integrating publishable sires’ international information from an international evaluation that did not include national data.

Table S10. Validation of the scenarios’ predictivity for direct EBV of offspring of publishable sires and for maternal EBV of daughters of MGS with publishable EBV when international information are integrated on Scenario NAT_JAN_ and EBV_INT_ are computed using pedigree-based international evaluations ^a^.

| **Validation group ^b^** | **EBV** | **Scenario ^c^** | **ρ** | **LB**  **(GSD)** | **b_1_** | **R^2^_adj_** | **RMSE**  **(GSD)** |
| --- | --- | --- | --- | --- | --- | --- | --- |
| Offspring of sires with publishable direct EBV  (n = 1,016) | Direct EBV | NAT_JAN_ | 0.86 | -0.11 | 0.89 | 0.73 | 0.24 |
|  |  | BLEND_JAN_ | 0.88 | -0.02 | 0.93 | 0.78 | 0.21 |
|  | Maternal EBV | NAT_JAN_ | 0.95 | 0.05 | 1.02 | 0.90 | 0.11 |
|  |  | BLEND_JAN_ | 0.94 | -0.01 | 1.01 | 0.88 | 0.12 |
| Offspring of sires  with publishable  direct and maternal EBV  (n = 60) | Direct EBV | NAT_JAN_ | 0.53 | -0.20 | 0.63 | 0.27 | 0.39 |
|  |  | BLEND_JAN_ | 0.67 | -0.07 | 0.81 | 0.44 | 0.35 |
|  | Maternal EBV | NAT_JAN_ | 0.87 | 0.07 | 0.95 | 0.76 | 0.18 |
|  |  | BLEND_JAN_ | 0.89 | -0.01 | 0.92 | 0.79 | 0.17 |
| Daughters of MGS with publishable direct and maternal EBV  (n = 740) | Direct EBV | NAT_JAN_ | 0.95 | -0.07 | 0.96 | 0.89 | 0.14 |
|  |  | BLEND_JAN_ | 0.99 | -0.02 | 0.97 | 0.98 | 0.06 |
|  | Maternal EBV | NAT_JAN_ | 0.93 | 0.03 | 0.97 | 0.87 | 0.14 |
|  |  | BLEND_JAN_ | 0.93 | -0.01 | 0.96 | 0.87 | 0.14 |

^a^ The EBV of the different scenarios are compared with pedigree-based EBV of scenario REF_APR_ (international evaluation including national data until April 2019 and foreign data until April 2019).

ρ: Pearson correlation of EBV, LB (GSD): level bias (in genetic standard deviations), b_1_: slope, R^2^_adj_: adjusted R^2^, RMSE (GSD): Root Mean Square Error (in genetic standard deviations).

^b^ Validation group = Offspring of publishable sires for direct EBV, and for direct and maternal EBV, with records in Italy born between January 2019 and April 2019. Daughters of MGS with publishable direct and maternal EBV: maternal-grand-sires’ daughters having recorded offspring in Italy born between January 2019 and April 2019.

^c^ Scenario: NAT_JAN_ = National evaluation using only national phenotypes prior to January 2019. BLEND_JAN_ = A blended national evaluation using national phenotypes as in NAT_JAN_ and integrating information of publishable sires from scenario INT_JAN_ (INT_JAN_ = international evaluation including national data prior to January 2019 and foreign data prior to January 2019).

Table S11. Validation of the scenarios’ predictivity for direct EBV of offspring of publishable sires and for maternal EBV of daughters of MGS with publishable EBV when international information are integrated on Scenario NAT_JAN_ and EBV_INT_ are computed using single-step international evaluations ^a^.

| **Validation group ^b^** | **EBV** | **Scenario ^c^** | **ρ** | **LB**  **(GSD)** | **b_1_** | **R^2^_adj_** | **RMSE**  **(GSD)** |
| --- | --- | --- | --- | --- | --- | --- | --- |
| Offspring of sires with publishable direct EBV  (n = 1,016) | Direct EBV | NAT_JAN_ | 0.85 | -0.12 | 0.89 | 0.73 | 0.24 |
|  |  | BLEND_JAN_ | 0.89 | -0.06 | 0.95 | 0.78 | 0.21 |
|  | Maternal EBV | NAT_JAN_ | 0.95 | 0.05 | 1.02 | 0.90 | 0.11 |
|  |  | BLEND_JAN_ | 0.95 | 0.01 | 1.02 | 0.90 | 0.12 |
| Offspring of sires  with publishable  direct and maternal EBV  (n = 60) | Direct EBV | NAT_JAN_ | 0.53 | -0.21 | 0.63 | 0.27 | 0.40 |
|  |  | BLEND_JAN_ | 0.65 | -0.11 | 0.80 | 0.41 | 0.36 |
|  | Maternal EBV | NAT_JAN_ | 0.87 | 0.07 | 0.95 | 0.76 | 0.18 |
|  |  | BLEND_JAN_ | 0.89 | 0.03 | 0.95 | 0.80 | 0.16 |
| Daughters of MGS with publishable direct and maternal EBV  (n = 740) | Direct EBV | NAT_JAN_ | 0.94 | -0.08 | 0.97 | 0.88 | 0.15 |
|  |  | BLEND_JAN_ | 0.99 | -0.04 | 1.00 | 0.97 | 0.07 |
|  | Maternal EBV | NAT_JAN_ | 0.93 | 0.03 | 0.97 | 0.87 | 0.14 |
|  |  | BLEND_JAN_ | 0.93 | 0.01 | 0.96 | 0.87 | 0.14 |

^a^ The EBV of the different scenarios are compared with single-step EBV of scenario REF_APR_ (international evaluation including national data until April 2019 and foreign data until April 2019).

ρ: Pearson correlation of EBV, LB (GSD): level bias (in genetic standard deviations), b_1_: slope, R^2^_adj_: adjusted R^2^, RMSE (GSD): Root Mean Square Error (in genetic standard deviations).

^b^ Validation group = Offspring of publishable sires for direct EBV, and for direct and maternal EBV, with records in Italy born between January 2019 and April 2019. Daughters of MGS with publishable direct and maternal EBV: maternal-grand-sires’ daughters having recorded offspring in Italy born between January 2019 and April 2019.

^c^ Scenario: NAT_JAN_ = National evaluation using only national phenotypes prior to January 2019. BLEND_JAN_ = A blended national evaluation using national phenotypes as in NAT_JAN_ and integrating information of publishable sires from scenario INT_JAN_ (INT_JAN_ = international evaluation including national data prior to January 2019 and foreign data prior to January 2019).

Table S12. Validation of the GOLD ^a^ scenario’s adequacy for direct and maternal EBV of publishable sires when EBV_INT_ are computed using pedigree-based international evaluations ^b^.

|  | **Validation group ^c^** | **ρ** | **LB**  **(GSD)** | **b_1_** | **R^2^_adj_** | **RMSE**  **(GSD)** |
| --- | --- | --- | --- | --- | --- | --- |
| Direct  EBV | Domestic (≥ 15 off)  (n = 1,382) | 0.99 | -0.03 | 1.01 | 0.98 | 0.07 |
|  | Domestic (< 15 off)  (n = 94) | 0.97 | -0.13 | 1.03 | 0.93 | 0.16 |
|  | Foreign  (n = 3,470) | 0.97 | -0.14 | 1.14 | 0.94 | 0.16 |
| Maternal  EBV | Domestic (≥ 15 off)  (n=491) | 0.98 | -0.01 | 1.02 | 0.97 | 0.09 |
|  | Domestic (< 15 off)  (n=51) | 0.86 | -0.03 | 0.87 | 0.74 | 0.22 |
|  | Foreign  (n=1,165) | 0.81 | 0.03 | 0.58 | 0.65 | 0.18 |

^a^ GOLD: A blended evaluation using national phenotypes as in NAT_APR_ and integrating information of publishable sires from an international evaluation including only foreign data prior to January 2019. NAT_APR_: national evaluation without integration.

^b^ The EBV of the different scenarios are compared with single-step EBV of scenario REF_APR_trunc_ (international evaluation including national data until April 2019 and foreign data prior to January 2019).

ρ: Pearson correlation of EBV, LB (GSD): level bias (in genetic standard deviations), b_1_: slope, R^2^_adj_: adjusted R^2^, RMSE (GSD): Root Mean Square Error (in genetic standard deviations).

^c^ Validation group = Domestic (≥ 15 off): publishable sires with at least 15 recorded offspring in Italy, Domestic (< 15 off): publishable sires with less than 15 recorded offspring in Italy, and Foreign: publishable sires with no recorded offspring in Italy.

Table S13. Validation of the GOLD ^a^ scenario’s predictivity for direct EBV of offspring of publishable sires and for maternal EBV of daughters of MGS with publishable EBV when EBV_INT_ are computed using pedigree-based international evaluations ^b^.

| **Validation**  **group ^c^** | **EBV** | **ρ** | **LB**  **(GSD)** | **b_1_** | **R^2^_adj_** | **RMSE**  **(GSD)** |
| --- | --- | --- | --- | --- | --- | --- |
| Offspring of sires with publishable direct EBV  (n = 1,016) | Direct EBV | 0.99 | -0.05 | 1.01 | 0.99 | 0.05 |
|  | Maternal EBV | 0.99 | 0.00 | 1.04 | 0.98 | 0.06 |
| Offspring of sires with publishable direct and maternal EBV  (n = 60) | Direct EBV | 1.00 | -0.05 | 1.02 | 0.99 | 0.05 |
|  | Maternal EBV | 0.98 | -0.02 | 0.97 | 0.97 | 0.07 |
| Daughters of MGS with publishable direct and maternal EBV (n = 740) | Direct EBV | 0.99 | -0.03 | 1.01 | 0.98 | 0.06 |
|  | Maternal EBV | 0.99 | 0.00 | 1.02 | 0.98 | 0.05 |

^a^ GOLD: A blended evaluation using national phenotypes as in NAT_APR_ and integrating information of publishable sires from an international evaluation including only foreign data prior to January 2019. NAT_APR_: national evaluation without integration

^b^ The EBV of the different scenarios are compared with single-step EBV of scenario REF_APR_ (international evaluation including national data until April 2019 and foreign data until April 2019).

ρ: Pearson correlation of EBV, LB (GSD): level bias (in genetic standard deviations), b_1_: slope, R^2^_adj_: adjusted R^2^, RMSE (GSD): Root Mean Square Error (in genetic standard deviations).

^b^ Validation group = Offspring of publishable sires for direct EBV, and for direct and maternal EBV, with records in Italy born between January 2019 and April 2019. Daughters of MGS with publishable direct and maternal EBV: maternal-grand-sires’ daughters having recorded offspring in Italy born between January 2019 and April 2019.

Table S14. Validation of the GOLD ^a^ scenario’s adequacy for direct and maternal EBV of publishable sires when EBV_INT_ are computed using single-step international evaluations ^b^.

|  | **Validation group ^c^** | **ρ** | **LB**  **(GSD)** | **b_1_** | **R^2^_adj_** | **RMSE**  **(GSD)** |
| --- | --- | --- | --- | --- | --- | --- |
| Direct  EBV | Domestic (≥ 15 off)  (n = 1,382) | 0.98 | -0.14 | 1.01 | 0.96 | 0.11 |
|  | Domestic (< 15 off)  (n = 94) | 0.93 | -0.25 | 1.02 | 0.87 | 0.23 |
|  | Foreign  (n = 3,470) | 0.90 | -0.20 | 1.04 | 0.81 | 0.27 |
| Maternal  EBV | Domestic (≥ 15 off)  (n=491) | 0.97 | 0.05 | 1.02 | 0.94 | 0.12 |
|  | Domestic (< 15 off)  (n=51) | 0.79 | 0.06 | 0.84 | 0.61 | 0.27 |
|  | Foreign  (n=1,165) | 0.74 | 0.00 | 0.53 | 0.54 | 0.21 |

^a^ GOLD: A blended evaluation using national phenotypes as in NAT_APR_ and integrating information of publishable sires from an international evaluation including only foreign data prior to January 2019. NAT_APR_: national evaluation without integration

^b^ The EBV of the different scenarios are compared with single-step EBV of scenario REF_APR_trunc_ (international evaluation including national data until April 2019 and foreign data prior to January 2019).

ρ: Pearson correlation of EBV, LB (GSD): level bias (in genetic standard deviations), b_1_: slope, R^2^_adj_: adjusted R^2^, RMSE (GSD): Root Mean Square Error (in genetic standard deviations).

^c^ Validation group = Domestic (≥ 15 off): publishable sires with at least 15 recorded offspring in Italy, Domestic (< 15 off): publishable sires with less than 15 recorded offspring in Italy, and Foreign: publishable sires with no recorded offspring in Italy.

Table S15. Validation of the GOLD ^a^ scenario’s predictivity for direct EBV of offspring of publishable sires and for maternal EBV of daughters of MGS with publishable EBV when EBV_INT_ are computed using single-step international evaluations ^b^.

| **Validation**  **group ^b^** | **EBV** | **ρ** | **LB**  **(GSD)** | **b_1_** | **R^2^_adj_** | **RMSE**  **(GSD)** |
| --- | --- | --- | --- | --- | --- | --- |
| Offspring of sires with publishable direct EBV  (n = 1,016) | Direct EBV | 0.97 | -0.11 | 1.02 | 0.95 | 0.11 |
|  | Maternal EBV | 0.96 | 0.04 | 1.06 | 0.92 | 0.10 |
| Offspring of sires with publishable direct and maternal EBV  (n = 60) | Direct EBV | 0.97 | -0.11 | 1.04 | 0.95 | 0.11 |
|  | Maternal EBV | 0.98 | 0.02 | 1.01 | 0.96 | 0.07 |
| Daughters of MGS with publishable direct and maternal EBV (n = 740) | Direct EBV | 0.97 | -0.09 | 1.03 | 0.93 | 0.11 |
|  | Maternal EBV | 0.97 | 0.03 | 1.02 | 0.95 | 0.09 |

^a^ GOLD: A blended evaluation using national phenotypes as in NAT_APR_ and integrating information of publishable sires from an international evaluation including only foreign data prior to January 2019. NAT_APR_: national evaluation without integration

^b^ The EBV of the different scenarios are compared with single-step EBV of scenario REF_APR_ (international evaluation including national data until April 2019 and foreign data until April 2019).

ρ: Pearson correlation of EBV, LB (GSD): level bias (in genetic standard deviations), b_1_: slope, R^2^_adj_: adjusted R^2^, RMSE (GSD): Root Mean Square Error (in genetic standard deviations).

^b^ Validation group = Offspring of publishable sires for direct EBV, and for direct and maternal EBV, with records in Italy born between January 2019 and April 2019. Daughters of MGS with publishable direct and maternal EBV: maternal-grand-sires’ daughters having recorded offspring in Italy born between January 2019 and April 2019.
