# Supplementary material for: Integration of beef cattle international pedigree and genomic estimated breeding values into national evaluations, with an application to the Italian Limousin population
Source: Genet Sel Evol. 2023 Jun 12;55:41. doi: 10.1186/s12711-023-00813-2 (PMC10258954; doi:10.1186/s12711-023-00813-2)
Supplement: Supplementary file 2 — Additional file 2. R functions to compute ERC, DRP, dERC* and DRP* (also available at https://github.com/bonifazi/Integration_EBV_and_GEBV). [file 12711_2023_813_MOESM2_ESM.docx]

# Additional file 2

R functions to compute ERC, DRP, dERC* and DRP* (also available at <https://github.com/bonifazi/Integration_EBV_and_GEBV>).

##########################################################################################

# R functions to compute ERC and DRP, and to compute dERC* and DRP*.

# Two R packages are required: *‘dplyr’* (for data manipulation) and *‘assertthat’* (for arguments’ type check).

# See the R functions documentation for input, output and usage description.

# Use the *‘docstring’* R package to view the help documentation of each function by typing for instance:

# *‘docstring(fun = compute_ERC_and_DRP)’*.

##########################################################################################

compute_DRP_adj <- function(nat_obj, int_obj) {

########################## DOCUMENTATION #####################################

#' Calculate adjusted ERC and adjusted DRP

#'

#' @description This function calculates adjusted effective record contributions (ERC*)

#' and adjusted de-regressed proofs (DRP*) for all animals in a given data.frame

#'

#' @param nat_obj (data.frame) R data.frame with the national data.

#' NOTE! these column names must be present:

#' (character) AID. animal ID.

#' (double) dERC. animal dERC.

#' (double) DRP. animal DRP.

#'

#' @param int_obj (data.frame) R data.frame with the international data.

#' NOTE! these column names must be present:

#' (character) AID. animal ID.

#' (double) dERC. animal dERC.

#' (double) DRP. animal DRP.

#'

#' @usage compute_DRP_adj(nat_obj, int_obj)

#'

#' @return

#' An R data.frame with ERC* and DRP* (*=adjusted) next to columns provided

#' as inputs and intermediate ones computed by this function.

#' Columns where if-conditions are applied are returned for post-checks and

#' have suffix "_fix" (see details).

#' AID column is used to merge nat_obj and int_obj. Columns of obj_nat are returned with

#' suffix "_nat". Column of obj_int have suffix "_int".

#'

#' @details Adjustment performed using formula from (Vandenplas and Gengler 2014):

#'

#' DRP* = (dERC_int*DRP_int) - (dERC_nat*DRP_nat) / dERC*

#'

#' where:

#' dERC* = (dERC_int-dERC_nat), and

#' dERC = ERC - ERC_pa

#'

#' NOTE: if-conditions implemented:

#' 1) if dERC_nat <= 0, then dERC_nat_fix = 0 and DRP_nat_fix = 0.

#' This avoids, DRP_nat to be 0 or +Inf/-Inf, which otherwise would lead to Nan in DRP*.

#'

#' 2) if dERC_int <= 0, then dERC_int_fix = 0 and DRP_int_fix = 0.

#' This harmonize and avoids same isses as with if-condition 1).

#'

#' 3) if dERC* <= 0, then dERC* = 0 and DRP* = 0. This avoids:

#' when dERC* = 0 a division by 0 in the formula which would lead to DRP* to +Inf or -Inf.

#' when dERC* < 0 which give issues when solving MME with DRP* and dERC*.

#'

#' NOTE: Columns where if-conditions were applied are returned for

#' post-checks and have suffix "_fix".

#'

#' @references

#' - Vandenplas J, Gengler N. Innovative algorithms to combine phenotypic,

#' genealogical and genomic information originating from diverse sources. 2014. PhD thesis.

#' Accessed 2018 Aug 10. https://api.semanticscholar.org/CorpusID:131179270

#'

#' @examples

#' compute_DRP_adj(nat_obj=NAT_Jan_DRP$direct, int_obj=INT_Jan_DRP_PBLUP$direct)

#'

#' @importFrom

#' dplyr assertthat

############################### COMPUTATION ###################################

# check that args column names are correct

assertthat::assert_that(

"AID" %in% colnames(nat_obj), "dERC" %in% colnames(nat_obj), "DRP" %in% colnames(nat_obj),

"AID" %in% colnames(int_obj), "dERC" %in% colnames(int_obj), "DRP" %in% colnames(int_obj),

msg = "Column names must contain 'AID', 'dERC' and 'DRP' for both nat_obj and int_obj."

)

# A) Merge int and nat dERC and DRP info using AID as merging column

data.tmp <- int_obj %>%

left_join(nat_obj, by=c("AID"), suffix=c("_int", "_nat"))

# B) Fix (DRP_nat & dERC_nat) and (DRP_int & dERC_int) to 0 when dERC_nat and dERC_int (respectively) are <= 0

data_fix.tmp <- data.tmp %>%

# if-condition n. 1) Set DRP_nat & dERC_na to 0 if dERC_nat <= 0

mutate(dERC_nat_fix = if_else(dERC_nat <= 0, true = 0, false = dERC_nat )) %>% # part A) if dERC_nat <= 0, then dERC_nat_fix = 0, else dERC_nat_fix = dERC_nat

mutate( DRP_nat_fix = if_else(dERC_nat <= 0, true = 0, false = DRP_nat )) %>% # part B) if dERC_nat <= 0, then DRP_nat_fix = 0, else DRP_nat_fix = DRP_nat

# if-condition n. 2) Set DRP_int & dERC_int to 0 if dERC_int <= 0

mutate(dERC_int_fix = if_else(dERC_int <= 0, true = 0, false = dERC_int )) %>% # part A) if dERC_int <= 0, then dERC_int_fix = 0, else dERC_int_fix = dERC_int

mutate( DRP_int_fix = if_else(dERC_int <= 0, true = 0, false = DRP_int )) # part B) if dERC_int <= 0, then DRP_int_fix = 0, else DRP_int_fix = DRP_int

# C) compute dERC* and DRP*

DRP_star.tmp <- data_fix.tmp %>%

mutate(dERC_adj = (dERC_int_fix - dERC_nat_fix) ) %>% # compute dERC* using fixed dERC_int and fixed dERC_nat

# if-condition n. 3) Set dERC* and DRP* to 0 if dERC* <= 0

mutate(dERC_adj_fix = if_else(dERC_adj <= 0, true = 0, false = dERC_adj)) %>% # part A) if dERC_adj <= 0, then dERC_adj_fix = 0, else dERC_adj_fix = dERC_adj

# compute DRP*

mutate(DRP_adj = if_else(dERC_adj <= 0, true = 0, false = ( # if-condition n.3) part B) if dERC_adj_fix <= 0, then DRP_adj=0, else use formula for DRP*

((dERC_int_fix*DRP_int_fix)-(dERC_nat_fix*DRP_nat_fix))/dERC_adj_fix) # compute DRP* = (dERC_int*DRP_int)-(dERC_nat*DRP_nat)/dERC*.

# NOTE: numerator use fixed (both NAT and INT) dERC and DRP

)

)

# check that DRP_star != +- Inf

if( all(is.infinite(DRP_star.tmp$DRP_adj)) ) {

stop("ERROR: Adjusted DRP contain +Inf and/or -Inf values", call. = T)}

return(DRP_star.tmp)

}

compute_ERC_and_DRP <- function(data, method_ERC, method_DRP, sigma2_a = NA, sigma2_e = NA, h2 = NA) {

#################### DOCUMENTATION ###########################################

#' Compute ERC and DRP

#'

#' @description This function computes de-regressed proofs (DRP) and effective

#' records contributions (ERC)and return them as an additional column to

#' provided data. Intermediate computed values ( sire_ebv, sire_rel, dam_ebv,

#' dam_rel, PA_ebv,PA_rel, ERC, ERC_PA, dERC ) are also returned as additional

#' columns.

#' NOTE: sire and dam ebv and rel are included in the data as animal_id (see details).

#'

#' @param data (data.frame) R data.frame with these columns (NOTE: column order is important!):

#' column 1) (character) animal_id

#' column 2) (character) sire_id

#' column 3) (character) dam_id

#' column 4) (numeric) EBV

#' column 5) (numeric) REL

#'

#' @param method_ERC (character) method used to compute ERC: either "RatioVariances" or "h2" (see details)

#' @param method_DRP (character) method used to compute DRP: either "VRetal2009" or "GRetal2009" (see details)

#' @param sigma2_a (numeric) genetic variance. To be provided only if lambda_method is "RatioVariances" (see details).

#' @param sigma2_e (numeric) residual variance. To be provided only if lambda_method is "RatioVariances" (see details).

#' @param h2 (numeric) heritability. To be provided only if lambda_method is "h2" (see details).

#'

#' @usage

#' compute_ERC_and_DRP(data=df, method_ERC="RatioVariances", method_DRP = "VRetal2009", sigma2_a=20, sigma2_e=40)

#' compute_ERC_and_DRP(data=df, method_ERC="h2", method_DRP = "GRetal2009", h2=0.30)

#'

#' @details

#' NOTE: An assumption is that sires' and dams' EBV and REL are included in the

#' provided data.frame as individuals themselves.

#' Missing parents get EBV = 0 and REL = 0.

#'

#' De-regressed proof (DRP) computed as Garrick et al. (GSE, 2009):

#' DRP = PA_EBV + (EBV - PA_EBV) * REL_own+prog

#'

#' De-regressed proof (DRP) computed as VanRaden et al. (JDS, 2009):

#'

#' DRP = PA_EBV + (EBV - PA_EBV) * (ERC/dERC)

#'

#' where:

#' - ERC = Effective Record Contribution (ERC) of animal i (computed following

#' Calus et. al, (JDS, 2016), see R function compute_ERC for more details).

#' - PA_EBV = Parent Average EBV computed as (sire_ebv + dam_ebv)/2

#' - dERC = De-regressed ERC computed following Calus et. al. (JDS, 2016),

#' i.e., dERC = ERC - ERC_PA, with ERC_PA computed from PA_REL and

#' PA_REL=(sire_rel + dam_rel)/4

#' - REL_own+prog = Reliability of own performance and progeny, computed from dERC

#' as described in Calus et. al. (JDS, 2016).

#'

#' @references

#' - Garrick, D.J., Taylor, J.F., Fernando, R.L., 2009. Deregressing estimated

#' breeding values and weighting information for genomic regression analyses.

#' Genet. Sel. Evol. 41, 55. https://doi.org/10.1186/1297-9686-41-55

#'

#' - VanRaden PM, Van Tassell CP, Wiggans GR, Sonstegard TS, Schnabel RD,

#' Taylor JF, Schenkel FS. Invited Review: Reliability of genomic predictions

#' for North American Holstein bulls. J Dairy Sci. 2009;92:16–24.

#' https://doi.org/10.3168/jds.2008-1514

#'

#' - Calus MPL, Vandenplas J, ten Napel J, Veerkamp RF.

#' Validation of simultaneous deregression of cow and bull breeding values

#' and derivation of appropriate weights. J Dairy Sci. 2016;99:6403–19.

#' https://doi.org/10.3168/jds.2016-11028

#'

#' @returns an R data.frame with the following columns:

#'

#' - sire_ebv and dam_ebv = (2 columns) EBV of sire and dam

#' - sire_rel and dam_rel = (2 columns) REL of sire and dam

#' - pa_ebv = Parent Average EBV

#' - pa_rel = Parent Average REL

#' - ERC = Effective Records Contribution for the animal, i.e. computed from REL provided

#' - ERC_PA = Effective Records Contribution for the parents, i.e. computed from PA_REL

#' - dERC = De-regressed ERC

#' - DRP = De-regressed proof

#' - sigma2a and sigma2e = (2 columns) if method_ERC="RatioVariances"

#' - h2 = if method_ERC = "h2"

#' - REL_from_dERC = if method_DRP = "GRetal2009"

#'

#' @importFrom

#' assertthat, dplyr, compute_ERC, convert_ERC_to_REL

#'

##############################################################################

# **** Start computations **** ###############################################

# Check input arguments type

assertthat::assert_that(

is.character(data[,1]), is.character(data[,2]), is.character(data[,3]),

is.numeric(data[,4]), is.numeric(data[,5]),

is.na(sigma2_a) || is.numeric(sigma2_a) && length(sigma2_a)==1,

is.na(sigma2_e) || is.numeric(sigma2_e) && length(sigma2_e)==1,

is.na(h2) || is.numeric(h2) && length(h2)==1

)

# 1) Store input colnames and rename cols (for dplyr use) --------------------

original_colnames <- colnames(data) # store input colnames

colnames(data) <- c("aid", "sire", "dam", "ebv", "rel") # rename colnames

# 2) Expand data cols with sire and dam EBV and REL, and compute PA_EBV and PA_REL -----------------

data_2 <- data %>%

left_join(data %>% select(sire = aid, sire_ebv = ebv, sire_rel = rel), by = "sire") %>% # expand df with sire ebv and rel

left_join(data %>% select( dam = aid, dam_ebv = ebv, dam_rel = rel), by = "dam") %>% # expand df with dam ebv and rel

replace_na(list(sire_ebv = 0, dam_ebv = 0, sire_rel = 0, dam_rel = 0)) %>% # if NA's present due to missing parents info replace NA's with 0 for both EBV and REL

mutate( # compute PA EBV and REL

pa_ebv = (sire_ebv + dam_ebv) / 2, # Parent Average EBV = (sire_EBV + dam_EBV) / 2

pa_rel = (sire_rel + dam_rel) / 4, # Parent Average REL = (sire_REL + dam_REL) / 4

)

# 3) Compute animal ERC from animal REL ----------------------

# apply ERC method as defined by user

if (method_ERC == "RatioVariances") {

animal_ERC <- data_2 %>%

select(aid, rel) %>% # select animal REL

compute_ERC(data = ., lambda_method = method_ERC,

sigma2_a = sigma2_a, sigma2_e = sigma2_e) # compute ERC

} else if (method_ERC == "h2") {

animal_ERC <- data_2 %>%

select(aid, rel) %>% # select animal REL

compute_ERC_h2(data = ., h2 = h2) # compute ERC

}

# 4) Compute ERC_PA (ERC from PA_REL) --------------------------------------------------

if (method_ERC == "RatioVariances") {

PA_ERC <- data_2 %>%

select(aid, pa_rel) %>% # select parent average REL

compute_ERC(data = ., lambda_method = method_ERC,

sigma2_a = sigma2_a, sigma2_e = sigma2_e) %>% # compute ERC_PA

rename(ERC_PA = ERC)

} else if (method_ERC == "h2") {

PA_ERC <- data_2 %>%

select(aid, pa_rel) %>% # select parent average REL

compute_ERC_h2(data = ., h2 = h2) %>% # compute ERC_PA

rename(ERC_PA = ERC)

}

# 5) Add previously computed animal ERC and ERC_PA to the data, and compute dERC (i.e. ERC - ERC_PA) ---------

data_3 <- data_2 %>%

left_join(animal_ERC, by = c("aid", "rel")) %>% # add ERC on animal

left_join(PA_ERC, by = c("aid", "pa_rel")) %>% # add PA_ERC

mutate(dERC = ERC - ERC_PA) # compute dERC (ERC - ERC_PA)

# 6) Compute DRP --------------------------------------------------------------

# ___ 6.1) DRP method is VanRaden et al., 2009 ------------------------------------

if (method_DRP == "VRetal2009") {

# Formula VanRaden et al., 2009 is: DRP = PA_EBV + (EBV - PA_EBV) * (ERC/dERC)

data_4 <- data_3 %>%

mutate(DRP = pa_ebv + ((ebv - pa_ebv) * (ERC / dERC)) )

} else if (

# ___ 6.2) DRP method is Garrick et al., 2009 ------------------------------------

method_DRP == "GRetal2009") {

# First compute REL corresponding to the dERC using same ERC method

# used so far for ERC and ERC_PA (either h2 or RatioVariances)

if (method_ERC == "h2") {

data_4 <- data_3 %>%

mutate(REL_dERC = convert_ERC_to_REL(ERC = dERC, lambda_method = method_ERC, h2 = h2))

} else if (method_ERC == "RatioVariances") {

data_4 <- data_3 %>%

mutate(REL_dERC = convert_ERC_to_REL(ERC = dERC, lambda_method = method_ERC, sigma2_a = sigma2_a, sigma2_e = sigma2_e))

}

# Compute DRP using Garrick et al., 2009

# Formula Garrick et al., 2009 is: DRP = PA_EBV + (EBV - PA_EBV) / REL_from_dERC

data_4 <- data_4 %>% mutate( DRP = pa_ebv + ((ebv - pa_ebv) / REL_dERC) )

}

# 7) Add to output info sigma2_a and sigma2_e, or h2 based on "method_ERC" ---------

if (method_ERC == "RatioVariances") {

data_4$sigma2_a <- sigma2_a

data_4$sigma2_e <- sigma2_e

} else if (method_ERC == "h2") {

data_4$h2 <- h2

}

# 8) Replace colnames changed in 1) with original colnames ---------------------------------

colnames(data_4)[1:length(original_colnames)] <- original_colnames

return(data_4)

}

compute_ERC <- function(data, lambda_method, sigma2_a = NA, sigma2_e = NA, h2 = NA) {

#################### DOCUMENTATION ###########################################

#' Calculate ERC from reliabilities

#'

#' @description This function computes ERC and return them as an additional

#' column to provided obj.

#'

#' @param data (data.frame) R data.frame with 2 columns (NOTE: Column order is important!):

#' - column 1: (character) animal ID

#' - column 2: (numeric) animal REL

#' @param lambda_method (character) method used to define lambda, either "h2" or "RatioVariances" (see details).

#' @param sigma2_a (scalar numeric) genetic variance (default=NA). To be provided only if lambda_method is "RatioVariances" (see details).

#' @param sigma2_e (scalar numeric) residual variance (default=NA). To be provided only if lambda_method is "RatioVariances" (see details).

#' @param h2 (numeric) heritability (default=NA). To be provided only if lambda_method is "h2" (see details).

#'

#' @usage

#' compute_ERC(data=df, lambda_method="RatioVariances", sigma2_a=20, sigma2_e=40)

#' compute_ERC(data=df, lambda_method="h2", h2 = 0.30)

#'

#' @returns

#' Returns an R data.frame with ERC as an additional column.

#'

#' @details

#' ERC calculated following Calus et. al. (JDS, 2016) as:

#' ERC = lambda*(REL /(1-REL) ),

#' where:

#' lambda = sigma2_e/sigma2_a (when lambda_method = "RatioVariances")

#' or

#' lambda = (1-h2)/h2 (when lambda_method = "h2")

#'

#' @references

#' Calus MPL, Vandenplas J, ten Napel J, Veerkamp RF.

#' Validation of simultaneous deregression of cow and bull breeding values and

#' derivation of appropriate weights. J Dairy Sci. 2016;99:6403-19

#'

#' @importFrom

#' assertthat

#

##############################################################################

# **** Start computations **** ###############################################

# Check input arguments type

assertthat::assert_that(is.character(data[, 1]), is.numeric(data[, 2]), is.character(lambda_method),

is.na(sigma2_a) || is.numeric(sigma2_a) && length(sigma2_a) == 1,

is.na(sigma2_e) || is.numeric(sigma2_e) && length(sigma2_e) == 1,

is.na(h2) || is.numeric(h2) && length(h2) == 1

)

# simple check REL for provided REL (REL must be 0-1 bounded)

if (min(data[, 2]) < 0 | max(data[, 2] > 1)) {

stop("ERROR: error in REL column (column 2 in function compute_ERC).\nValues provided are not within 0-1 bound!\nDid you not provided REL in col 2?\nPossible EBV provided instead of REL.",

call.=T)

}

# compute lambda

if (lambda_method == "RatioVariances") {

lambda <- sigma2_e / sigma2_a # lambda = sigma2_e/sigma2_a

} else if (lambda_method == "h2") {

lambda <- (1 - h2) / h2 # lambda = (1-h2)/h2

}

# compute ERC

data$ERC <- lambda * (data[, 2] / (1 - data[, 2])) # ERC = lambda * [ REL / (1-REL) ]

return(data)

}

convert_ERC_to_REL <- function(ERC, lambda_method, sigma2_a = NA, sigma2_e = NA, h2 = NA) {

#################### DOCUMENTATION ###########################################

#' Convert ERC to REL

#'

#' @description This function converts Effective Records Contribution (ERC) in corresponding Reliabilities (REL)

#'

#' @param ERC (numeric) vector of ERC.

#' @param lambda_method (character) The method used to define lambda, either "h2" or "RatioVariances" (see details).

#' @param sigma2_a (numeric) genetic variance (default=NA). To be provided only if lambda_method is "RatioVariances" (see details).

#' @param sigma2_e (numeric) residual variance (default=NA). To be provided only if lambda_method is "RatioVariances" (see details).

#' @param h2 (numeric) heritability (default=NA). To be provided only if lambda_method is "h2" (see details).

#'

#' @usage

#' convert_ERC_to_REL(ERC=ERC_nat, lambda_method="RatioVariances", sigma2_a=20, sigma2_e=40)

#' convert_ERC_to_REL(ERC=ERC_nat, lambda_method="h2", h2=0.30)

#'

#' @returns

#' A vector of REL

#'

#' @details

#' REL is calculated as:

#'

#' REL = ERC/( ERC + lambda),

#' where

#' lambda = sigma2_e/sigma2_a, when lambda_method is "RatioVariances"

#' or

#' lambda = (1-h2)/h2, when lambda_method is "h2"

#'

#' @importFrom

#' assertthat

#

##############################################################################

# **** Start computations **** ###############################################

# Check input arguments type

assertthat::assert_that(is.numeric(ERC), is.character(lambda_method),

is.na(sigma2_a) || is.numeric(sigma2_a) && length(sigma2_a) == 1,

is.na(sigma2_e) || is.numeric(sigma2_e) && length(sigma2_e) == 1,

is.na(h2) || is.numeric(h2) && length(h2) == 1)

# compute lambda

if (lambda_method == "RatioVariances") {

lambda <- sigma2_e / sigma2_a # lambda = sigma2_e/sigma2_a

} else if (lambda_method == "h2") {

lambda <- (1 - h2) / h2 # lambda = (1-h2)/h2

}

# convert ERC into REL

REL <- ERC / (ERC + lambda)

if (min(REL) < 0) { # check REL are > 0 (this can happen when ERC provided are negative)

warning("Warning: there are negative REL (< 0). You may want to set these to 0.", call. = T)

}

if (max(REL) > 1) { # check REL is not > 1 (this must not happen)

stop("ERROR: there are REL > 1", call. = T)

}

return(REL)

}
